# Cervical Cancer Screening Programs in Europe: The Transition Towards HPV Vaccination and Population-Based HPV Testing

**DOI:** 10.3390/v10120729

**Published:** 2018-12-19

**Authors:** Andreas C. Chrysostomou, Dora C. Stylianou, Anastasia Constantinidou, Leondios G. Kostrikis

**Affiliations:** 1 Department of Biological Sciences, University of Cyprus, 1 University Avenue, Aglantzia 2109, Nicosia, Cyprus; Chrysostomou.C.Andreas@ucy.ac.cy (A.C.C.); Stylianou.C.Dora@ucy.ac.cy (D.C.S.); 2 Medical School, University of Cyprus, Shakolas Educational Center for Clinical Medicine, Palaios dromos Lefkosias Lemesou No.215/6 2029 Aglantzia, Nicosia, Cyprus; Constantinidou.Anastasia@ucy.ac.cy

**Keywords:** human papillomavirus, cervical cancer, cervical cytology, HPV test, HPV vaccination

## Abstract

Cervical cancer is the fourth most frequently occurring cancer in women around the world and can affect them during their reproductive years. Since the development of the Papanicolaou (Pap) test, screening has been essential in identifying cervical cancer at a treatable stage. With the identification of the human papillomavirus (HPV) as the causative agent of essentially all cervical cancer cases, HPV molecular screening tests and HPV vaccines for primary prevention against the virus have been developed. Accordingly, comparative studies were designed to assess the performance of cervical cancer screening methods in order to devise the best screening strategy possible. This review critically assesses the current cervical cancer screening methods as well as the implementation of HPV vaccination in Europe. The most recent European Guidelines and recommendations for organized population-based programs with HPV testing as the primary screening method are also presented. Lastly, the current landscape of cervical cancer screening programs is assessed for both European Union member states and some associated countries, in regard to the transition towards population-based screening programs with primary HPV testing.

## 1. Introduction

Cancer of the cervix uteri, more commonly known as cervical cancer, is an important public health concern. It was reported as the fourth most frequently occurring gynecological cancer, with an estimated worldwide incidence of 528,000 cases and 266,000 deaths in 2012 [1]. In Europe, an estimated 58,373 women are diagnosed annually with cervical cancer, and 24,404 of those die from this illness [2].

The incidence and mortality of cervical cancer, however, have been declining in developed countries due to the discovery of the Pap test in the 1940s, which enabled the prompt identification of morphological changes in the cervical epithelium [3]. The use of the Pap test in national screening programs can be dated back to the 1960s and 1970s [4], and it is still a cornerstone in the majority of current programs. Moreover, the International Agency for Research on Cancer (IARC) determined that the incidence of invasive cervical cancer can be reduced by at least 80% with the implementation of cervical cancer screening programs based on Pap test every three to five years for women of ages 35 to 64 [5,6,7,8,9].

Cervical cancer screening was revolutionized in the early 1980s by the discovery of human papillomaviruses (HPV) as the single causative agents of the disease. In 1983, HPV type 16 (HPV16) was first identified in DNA from a biopsy sample of invasive cancer of the cervix, and in the following years, HPVs were reported as the main causative agents of cervical cancer [10,11,12,13]. HPVs are small non-enveloped double-stranded DNA viruses with 221 officially characterized types, as of June 2018 [14]. These viruses have a genome of 8 kb that encodes early regulatory proteins (E1, E2, E5, E6, and E7), and late structural proteins (L1 and L2). HPVs are the most common sexually transmitted viruses [15,16,17]. According to estimates, approximately 80% of sexually active women will acquire the infection in their lifetime, and in the majority of cases (>90%), it will be a transient, asymptomatic infection cleared by the immune system in six months to two years [17,18,19]. Only after a persistent infection can HPV lead to low- and/or high-grade cervical intraepithelial neoplasia (CIN), which may eventually evolve to cervical cancer [17,20,21]. However, not all HPV types have been linked to cervical cancer. At least 12 types of HPV are epidemiologically classified as oncogenic, high-risk (hr) types (HPV16/18/31/35/39/45/51/52/56/58/66/68), which cause more than 97% of cervical cancer cases, while low-risk (lr) types (HPV6/11/40/42/43/44/54/61/72) are linked to anogenital warts and laryngeal papillomas [16,22,23,24]. The aforementioned HPV16 and HPV18 are the most commonly occurring hrHPV types, and cause approximately 70% of cervical cancers (~50% HPV16, ~20% HPV18) [17,25,26]. The elucidation of the etiological role of HPV has altered the landscape of cervical cancer screening in more ways than one. The fact that cervical cancer is primarily attributable to a single infectious agent enabled the development of new more sensitive HPV-based screening tests for secondary prevention of cervical cancer and three vaccines against HPV, which are utilized for primary prevention.

This review focuses on the available tests and strategies, which are currently employed for screening and prevention of HPV infection and cervical cancer. Furthermore, in accordance with recommendations specified in the recent European Guidelines, important aspects of screening programs necessary for the success and efficiency of such systems are highlighted. Finally, the current landscape of cervical cancer screening programs of member states of the European Union (E.U.) and some associated countries is reviewed.

## 2. Methodologies for Cervical Cancer Screening

### 2.1. Cervical Cytology

#### 2.1.1. Conventional Pap Test and Its Alternatives

Testing to identify anomalies in the cervix can be dated as far back as the early 19th century, when anatomists and pathologists of the time observed and studied the cytological changes derived from cervical and other genital neoplasms, as well as the woman’s menstrual cycle [27]. In the mid-1800s, the Irish physician Walter Hayle Walsh was the first to show that cancerous cells could be identified by microscopy [28,29]. In the early 20th century (1927), the Romanian physician Aurel Babeş detected the presence of cervical cancer by collecting cells from a woman’s cervix using a platinum loop and then observing them under a microscope. This process was the predecessor to what is known today as the Pap test [29].

With the invention of the Pap test in the 1940s, by George N. Papanicolaou and H.F. Traut, cervical cytology gained a robust and low-complexity method of screening for cervical cancer [30]. This process entails the exfoliation of cells from the cervix, which are then fixed, viewed under a microscope, and are subsequently morphologically interpreted. The staining method developed for this test offered a polychromatic definition of the nucleus and the features of the cytoplasm. The Pap test allows the assessment of nuclear chromatin alterations to discern whether necrosis occurred, the observation of the degree of cellular degeneration, and the distinction of the maturity of squamous epithelial cells [28,30,31].

Despite its widespread use as the primary cervical cancer screening method, the Pap test has some important limitations. The staining procedure of the conventional Pap test requires a considerable amount of time (20–30 min) and consumables [32]. The smearing process of the Pap test is also characterized by poor reproducibility and is vulnerable to obscuration by blood and mucus, imperfect fixation, and a non-uniform distribution of cells, thus causing errors in the detection and interpretation of the results. These issues can be attributed partly to the quality of sampling and can explain the broad range of sensitivity (30–87%) reported for the Pap test [33,34].

Consequently, to address the shortcomings of the Pap test, a number of derivative methods were developed, such as the UltraFast staining technique, the short-duration Papanicolaou stain, the REAP stain and the Enviro-Pap method [32,35,36,37,38,39]. These modifications significantly improved upon the conventional Pap smear performance in terms of speed and cost, and are also more environmentally friendly. The guiding principle of these enhancements was to improve at least one aspect of the smear without compromising the quality of the results [32,35,36,37,38].

#### 2.1.2. Liquid-Based Cytology

Another alternative method developed to address the shortcomings of the conventional Pap smear is liquid-based cytology (LBC). ThinPrep® Pap test (Hologic, Inc, Marlborough, MA) was the first LBC technique to be approved by the United States Food and Drug Administration (FDA) [34]. This method entails the collection of cells from the cervix, which are then transferred to a vial containing preservative solution instead of being fixed on a slide, thus enabling uniform distribution of the collected clinical material. Since only a portion of the sample is used for cytology, the rest can be employed for further testing, including HPV testing [33,40]. Presently, Thin Prep and SurePath (Becton Dickinson) are the two most frequently used LBC techniques. Several studies have shown significantly reduced numbers of unsatisfactory smears that would require repeat testing when LBC is used and some studies have also shown higher CIN detection rates compared to the conventional Pap test [34,41,42]. Conversely, other studies have questioned the advantages of LBC over the conventional Pap test and showed sensitivity less than or equal to that of the conventional Pap test [41,43,44,45,46].

#### 2.1.3. Visual Inspection by Acetic Acid and Visual Inspection with Lugol’s Iodine

Visual inspection by acetic acid (VIA) or with Lugol’s iodine (VILI) are two inexpensive screening methods frequently used in low-resource settings, with VIA being more widely used. These techniques are based on the fact that upon the application of acetic acid or Lugol’s iodine directly to the cervix, precancerous cervical lesions become discernible to the naked eye by both clinicians and nonclinicians. Although not perfect, both VIA and VILI have been reported to have acceptable specificity and sensitivity in low-resource settings [47,48,49].

Cervical cytology has undoubtedly played an important role in cervical cancer screening and continues to do so. However, it has inherited the limitation of being a morphological method requiring subjective interpretation by well-trained cytologists [11]. Despite continuous efforts to improve the performance of cervical cytology, its sensitivity is not optimal and the method still produces high numbers of borderline results, such as atypical squamous cells of undetermined significance (ASCUS, or ASC-US after the 2001 Bethesda Workshop) which, require further testing, tight follow-up and raise constant uncertainty for false negative results leading to over-referral to colposcopy and overtreatment [11,50,51,52]. 

### 2.2. HPV Testing

#### 2.2.1. Advantages and Limitations

In contrast to screening methods based on cytology, HPV testing does not rely on morphological interpretation and is based on the detection of HPV DNA, HPV mRNA or other viral markers. In the last two decades, HPV testing has become in several countries an invaluable part of clinical guidelines for cervical carcinoma screening, triage and follow-up after treatment [53]. As a general rule, HPV testing must be performed in appropriate, evidence-based contexts to maximize the benefit and reduce over-diagnosis. HPV testing for the identification of women at higher risk of developing cervical cancer, significantly differs from molecular testing for other medically relevant viruses, in that analytic sensitivity for the detection of HPV is not the prime driver of test performance. Unfortunately, the great majority of HPV tests currently on the market have high analytic sensitivity. Consequently, when they are used for agreed clinical indications they can yield a large number of clinically insignificant positives, resulting in more false referrals for colposcopy and biopsy, decreased correlation with the histological presence of disease, unnecessary treatment of healthy women and a consequent distrust of a positive result by the treating physician. Another important peculiarity of HPV testing for identification of women at higher risk for the development of cervical cancer is the need for balanced and artificially reduced coverage of the HPV testing types.

#### 2.2.2. Clinical Validation of HPV Tests

Taking into consideration these characteristics of HPV testing, when designing an HPV test to be used for agreed clinical indications, the ultimate sensitivity for the detection of precancerous lesions by inclusion of HPV types that are rarely associated with cervical cancer, must be carefully weighed against the potentially dramatic loss of clinical specificity when a particular HPV type (e.g., HPV53 and HPV66) is frequent in low-grade disease or in women without disease. In addition, it should always be taken into consideration that absolute reassurance following a negative cervical cancer screening test result is not achievable at any analytic sensitivity, because of a myriad of factors that are independent of the actual screening test performance, including operator error and poor cervical sampling. Thus, a cervical cancer screening program should adopt an HPV test for use as screening tool, only if it has been validated by demonstrating reproducible and consistently high sensitivity for CIN2+ and CIN3+ lesions, as well as minimal detection of clinically irrelevant, transient HPV infections [54,55]. There is a consensus in the HPV community that HPV tests (neither commercial nor in-house tests) that have not been clinically validated should not be used in clinical practice. HPV testing should be performed only on samples processed and analyzed in qualified laboratories, validated by authorized accreditation bodies and in compliance with international standards [54,55]. Laboratories involved in HPV-based screening should perform a minimum of 10,000 HPV tests per year [54,55].

Several comprehensive inventories of commercially available HPV tests were published in the last decade [56,57,58]. As of July 2018, at least 250 distinct commercial tests for detection of alpha HPVs and at least 230 variants of the original tests are available at the global market. Unfortunately, only a subset of commercial HPV tests has documented clinical performance for agreed indications for HPV testing in current clinical practice. For more than half of the HPV tests in the global market, no single publication in peer-reviewed literature can be identified [58]. In contrast to commercial kits for “classical” molecular microbiology targets, the great majority of HPV commercial tests currently on the market do not contain a sample extraction step and a number of them do not even mention recommended nucleic acid extraction methodology in their manufacturer’s instructions. Only a minority of HPV tests on the market have internal controls [58].

As a multitude of hrHPV tests are available, regular evaluation updates are essential to ensure their suitability for primary cervical cancer screening. A recent systematic review [59], listed the hrHPV DNA tests that were either validated through randomized trials showing a very low incidence of cervical cancer after a negative hrHPV DNA test [53,60] or fulfilling consensus-based international equivalence criteria based on cross-sectional data [8]. The international equivalence criteria are based on the non-inferior cross-sectional accuracy of a new HPV test versus one of the two benchmark comparator tests (GP5+/6+ PCR-EIA and/or Qiagen Hybrid Capture 2 HPV DNA Test) that have been validated in clinical trials and detect the same molecular targets, i.e., DNA of hrHPV types [61]. To fulfill the necessary criteria, the candidate test should demonstrate a relative sensitivity and specificity to detect CIN2+ compared to the standard comparator tests of more than 0.90 and 0.98 respectively, and show high inter- and intra-laboratory reproducibility [61]. Other potential cervical cancer screening tests based on other target molecules such as HPV mRNA, proteins or methylation markers cannot directly be considered equivalent and require additional evidence regarding their longitudinal effects, i.e., long-term safety [59]. The proper validation of HPV DNA tests, according to the international equivalence criteria can be problematic due to difficulties with obtaining an appropriate set of clinical specimens. The recently launched international framework “Validation of HPV Genotyping Tests (VALGENT)” facilitates the comparison and validation of HPV DNA tests by providing a set of samples obtained from women attending routine screening (1,000–1,300 samples) enriched with cytological abnormal samples (300 samples) [62]. In order to allow comparison with other HPV tests, each VALGENT panel includes a comparator assay that was previously clinically validated for cervical cancer screening purposes [62]. As of July 2018, only 14 commercial HPV assays (out of +480 HPV assays at the global market) can be considered as completely or partially validated for primary HPV-based cervical cancer screening [59,62]. The list includes four out of five HPV assays approved by US FDA: Hybrid Capture 2 (hc2) HPV DNA Test (Qiagen), cobas 4800 HPV Test (Roche), APTIMA HPV Assay (Hologic) and BD Onclarity HPV Assay (Becton Dickinson).

Since the performance of an HPV test may vary depending on the sample collection procedures and medium, regulatory approval in some settings requires validation of performance based on the choice of sample collection medium. Importantly, the validation of a pre-approved assay for use with a specific medium is a simpler process than de novo clinical validation of an HPV assay. It can be expected that several previously approved tests will eventually be validated for use with the most commonly used collection media [58].

It is worth mentioning that although we have an increasing understanding of which HPV tests are valid for HPV-based primary cervical cancer screening, given an internationally accepted and applied validation framework and published professional guidance, we do not have widely accepted equivalent metrics to judge the validity of HPV tests in other clinical settings, including post-treatment surveillance and the triage of low-grade abnormalities [58,59]. International efforts to create such validation guidelines will be of great benefit, since existing data show significant variation in commercially available tests being used in clinical settings that are not part of HPV-based primary cervical cancer screening programs.

## 3. Primary Cervical Cancer Prevention by HPV Vaccination

### 3.1. HPV Vaccines

The identification of HPV as the main etiological agent of cervical cancer, presented novel opportunities for the development of preventative modalities against cervical cancer [10]. With this knowledge, it became clear that stopping hr types of the virus from ever infecting should be explored as an option, in addition to the preexisting cervical cancer screening tests. Two decades long efforts culminated in 2006 with the approval of the first safe and efficacious HPV prophylactic vaccine [63]. The first vaccine that was approved was the quadrivalent Gardasil/Silgard, which targets HPV6, 11, 16, and 18 [64]. A year later, the bivalent Cervarix vaccine targeting HPV16 and 18 was approved, and more recently, the nonavalent Gardasil 9 vaccine, which targets HPV6, 11, 16, 18, 31, 33, 45, 52, and 58 was also approved [65]. All three of these vaccines target HPV16 and 18, and contain HPV L1 protein virus-like particles (VLPs) expressed in different cell types [16]. VLPs are morphologically and antigenically similar to native HPV virions, and because of the genomic similarity between different types of the virus, a certain degree of protection against HPV types not targeted by the vaccine, so called cross-protection, is also achieved. HPV vaccines elicit immunity through the production of high titers of anti-HPV IgG neutralizing antibodies, which block the entrance of the virus into the host cells [15]. The quadrivalent and nonavalent vaccines contain VLPs of two lrHPV types, 6 and 11, which are responsible for more than 90% of anogenital warts and laryngeal papillomas. Moreover, the nonavalent vaccine is targeted against the five types (HPV31, 33, 45, 52, 58) most frequently identified in cervical cancer after HPV16 and 18 [16,66,67]. Nonetheless, even with cross-protection and the increased number of HPV types covered by the nonavalent vaccine, HPV vaccines do not protect against all HPV types that cause cervical cancer [68,69]. 

### 3.2. Improving HPV Vaccination Coverage

Initially, all three HPV vaccines had been approved for a 3-dose series in order to generate sufficient and long-lasting protective immunity [70]. Currently, for all three vaccines two doses are recommended for persons starting the series before their 15th birthday and three dose schedule for those who start the series on or after their 15th birthday and for persons with certain immunocompromising conditions [71]. Decreasing the number of doses not only leads to reductions in overall cost, which is a concern (especially in low-income countries), but it also increases adherence to the program [71,72,73].

Despite their potency in providing protection against HPV infection, HPV vaccines are not therapeutic, as they are not effective in curing preexisting HPV infections [16]. Hence, current HPV vaccination programs are mainly targeted to both genders prior to coitarche, aiming to reduce the burden of cervical cancer and other HPV-related tumors, not only in vaccinated but also in unvaccinated individuals thanks to herd immunity [69]. As both genders are responsible for HPV transmission, both genders should be vaccinated to share the burden in reducing the risk of HPV-related disease, as well as to have equal access to direct vaccine benefits. It is becoming evident that only gender-neutral vaccination will lead to substantial control of HPV-related diseases both in women and men as well as maximizing prevention of cervical cancer, especially if vaccination coverage for girls in a particular program is not high. Current failure to implement gender-neutral HPV vaccination with high coverage in the great majority of countries looks like a missed unique public health opportunity [74,75]. However, even with the high protection against de novo HPV infections provided by HPV vaccines, successful cervical cancer prevention will still rely on screening for years to come [69] but future strategies will require substantial changes: longer screening intervals, exclusive use of HPV-based screening strategies as well as vaccination of older cohorts. An innovative strategy with the purpose of accelerating the reduction of cervical cancer incidence and mortality named “HPV-FASTER” has been recently proposed, with a generalized HPV vaccination campaign aimed at girls and women aged 9–45, paired with at least one HPV-based screening test at any age over 30 and eventual triage and diagnostic assessments among women who screen HPV-positive [76].

## 4. Cervical Cancer Screening Programs

### 4.1. Organization of Screening

With extensive knowledge of the biology of cervical cancer and with an arsenal of screening and prevention tools, the disease can be detected at an early enough stage to be curable. As a concept, the fundamental principles of cervical cancer screening can be dated back as far as the 1940s, before organized screening programs took place [4,77]. However, it was not until 1968 that Wilson and Jungner defined a set of criteria (comprehensively reviewed by Basu et. al. [47]) that not only helped to define whether a disease, such as cervical cancer, is eligible for screening but also influenced the development of better-thought-out screening programs. Undertakings of such magnitude, however, are no trivial tasks, since a number of prerequisites have to be accounted for before embarking on the implementation of such programs. The nature and parameters of the program, which are directly influenced and supported by scientific progress, must be established [47,78].

To this end, the first edition of the European Guidelines for Quality Assurance in Cervical Cancer Screening, published in 1993 [79], designated the principles for organized, population-based screening, with a number of countries adhering to this recommendation [80]. The supplements of the second edition of the European Guidelines for Quality Assurance in Cervical Cancer Screening of 2015 (the original volume of the second edition was published in 2008) emphasize the importance of the implementation of an organized, population-based cervical cancer screening program with a call/recall invitation system in order to take full advantage of the benefits of screening and discuss the key aspects of this type of organization in considerably increased detail [55,80]. Such a program should have a national/regional team that directs the implementation of guidelines, rules and procedures. This team would also be responsible for quality assurance to monitor and to guarantee that all levels of the process are performed sufficiently. This responsibility includes the management and coordination of the call/recall system, testing and diagnosis, as well as follow-up after positive test results. Furthermore, quality assurance procedures call for attention to training personnel, evaluating performance, auditing and monitoring, and reviewing the impact of the program on the burden of disease. The latter is facilitated by the population-based nature of the program, which is characterized by the identification and personal invitation of each member of the targeted population eligible for screening [6,81,82]. 

In contrast to organized population-based screening, opportunistic screening depends on the initiative of the individual woman and/or her doctor. This type of screening often results in high coverage only in certain parts of the population, which are screened frequently, while other parts of the population, usually with a lower socioeconomic status, exhibit lower coverage. This situation results in uneven coverage with heterogeneous quality, limited effectiveness, and reduced cost-effectiveness, as well as difficulty in monitoring the population [81]. 

Thus, as the European Guidelines recommend, a program with an organized population-based nature may substantially improve the accessibility and equity of screening access while simultaneously improving effectiveness and cost-effectiveness [6,81]. The key factors to be specified within such a program are the target age, screening intervals, and screening algorithm. The latter refers to the primary screening test and the subsequent management of results at each step of the algorithm.

### 4.2. Primary Screening Tests and Specifications

#### 4.2.1. Primary Cytology Testing

Three options that are currently in use for primary cervical cancer screening, are cytology, HPV testing, and cotesting [83]. Cytology-based testing has been used for primary screening for more than half a century and is currently employed by the majority of screening programs in Europe. However, it was implemented in screening programs in the 1960s-70s without being assessed in RCTs [47]. As described earlier in this review, cytology-based testing has various technical characteristics that affect its standing at the forefront of screening. It has undoubtedly proven its impact on reducing cervical cancer morbidity and mortality, especially in organized settings [84]. However, the low sensitivity of the technique, the requirement for high-quality diagnostic facilities, the high costs needed to sustain the infrastructure, and the need for highly trained personnel are important issues that have brought primary cytology screening under intense scrutiny for the past twenty years [85,86]. To maintain the accuracy and performance of cervical cytology, short intervals between screenings are required, which implies the performance of an increased number of tests and as such it can be costly [83]. Another factor that is already affecting the performance of cytology as a tool for primary screening is a reduced population burden of HPV due to HPV vaccination. The specificity of cytology, the main hallmark of the method, is decreasing in countries with high HPV coverage due to the dramatic population reduction of high-grade lesions as a result of HPV vaccination. Furthermore, since the current vaccines do not cover all HPV types causing cervical abnormalities, an increase in proportion of minor abnormalities caused by less carcinogenic HPV types is also expected, which in turn will further lower the once very high positive predictive value (PPV) of cytology [84,87]. As the population prevalence of hrHPVs and consequently CIN2/3 will decrease, screening modalities with higher sensitivity like HPV testing will clearly perform better at the population level. 

#### 4.2.2. Primary HPV Testing

The development of clinically validated HPV tests, which are more accurate and sensitive than primary cytology testing, has recently caused a paradigm shift. According to the European Guidelines, as well as the World Health Organization (WHO), HPV testing is now proposed as the primary screening tool for cervical cancer [55,88]. HPV testing is characterized by high clinical sensitivity, a high negative predictive value (NPV), objectiveness, low training requirements, reproducibility and a high throughput capacity [47,88,89]. HPV-based screening requires longer screening intervals than cytology-based screening since progression to cancer occurs years after an infection with hrHPV. Based on these facts, the European Guidelines recommend a five-year screening interval for HPV testing, which may be extended up to 10 years depending on the age and screening history of the patient [47,55,90,91]. Longer screening intervals contribute to less expensive programs, as well as providing a longer duration of “peace of mind” when women test negative in comparison to cytology-negative women [47,91]. Another factor that is expected to help establish primary HPV screening as a more cost-effective option is HPV vaccination. In a study performed to evaluate the effectiveness and cost-effectiveness of cervical cancer prevention scenarios, the most cost-effective strategy was the combination of preadolescent vaccination with an organized screening program, using primary HPV testing every five years with cytology triage [92]. In this regard, partial HPV genotyping may be worth employing either as part of primary HPV screening, which would entail using an HPV assay with genotyping capabilities, or as triage. This approach would not only help with the management of positive HPV cases but would also enable the direct monitoring of the downstream effects of vaccination [83,84,91,93].

When deciding at what age to start HPV-based screening it is important to take into account the natural history of HPV infection in order to avoid unnecessary follow-up and/or overtreatment of women with only transient HPV infections [47,94]. Thus, the European Guidelines recommend against primary HPV screening before the age of 30 and are in favor of screening starting at the age of 35, especially in a setting without prior cytology screening implemented. However, there is insufficient evidence to promote or restrict the start of HPV-based screening between the ages of 30–34. Conversely, in a region or country where primary cytology screening is running well, the policy-makers of the program may decide to implement primary HPV testing beginning at the recommended age of 30 or 35, while also maintaining their current cytology-based program from the ages of 20-30, at least until evidence shows otherwise. Nonetheless, the avoidance of screening prior to the age of 20 is recommended [53,55,91]. 

At the same time, setting the age to stop screening is important. The European Guidelines suggest that primary HPV screening could stop at the same age recommended for cytology, that is, at 60–65 years of age, provided that the most recent screening test was negative [55]. The reasoning for stopping screening at this age is due to the extremely low probability that an incident HPV infection will become persistent and that women will consequently develop cancer. Screening for a newly acquired HPV infection is therefore redundant and/or not cost-effective in women over 65 years with recent negative screen result(s) [95,96]. Furthermore, RCT data report significantly less CIN2/3 at ages 50–60 in comparison to 35–49 [60,97]. However, the European Guidelines state that current data are insufficient to select the optimal age to stop HPV primary testing, which was why the recommended age to stop screening for cytology was also kept for primary HPV testing. Nonetheless, it is important to note that cytology performs relatively poor at those ages, especially for postmenopausal women, in whom epithelial atrophy is commonly observed. Moreover, the cervical transformation zones of postmenopausal women are situated in the cervical canal, making the collection of material for cytological examination less accessible. Accordingly, cytology has low sensitivity for postmenopausal women, and screening can result in elevated false-positive results [95,98]. However, a recent Swedish study found that although HPV prevalence is relatively low in older women, there was still an increased risk for cervical dysplasia upon a second positive HPV screen test [98]. Furthermore, 30% of cervical cancer cases were still diagnosed in women older than 60, with a mortality as high as 70%. These findings, coupled with the non-optimal performance of cytology in older women, suggest the extension of the screening age as well as the need for more research [98].

#### 4.2.3. Primary HPV Cotesting

Cotesting combines the sensitivity of HPV testing with the specificity of cytology at the level of primary screening. Even though some non-European studies reported marginal superiority of cotesting over HPV-based screening alone, the European Guidelines recommend against cotesting at any given age because it is not substantially more effective than HPV testing and is considerably more costly [55,83,89]. 

### 4.3. Management of Women after Primary Screening

#### 4.3.1. Management of Women after a Positive HPV Primary Test Result

Having established HPV testing as the recommended primary screening method, the age range, and the screening intervals of a negative test, it is important to specify the management of positive results from primary testing. Triaging women with a positive HPV primary test result can compensate for the lower specificity that characterizes HPV testing. In this regard, the European Guidelines recommend the performance of cytology as the main triage test in order to manage the increased number of screen positives identified by primary HPV testing, which would otherwise lead to an excessive number of referrals to colposcopy. Thus, only women with both an HPV-positive result and cytological abnormalities are immediately referred for colposcopy. If the primary HPV test employs partial genotyping for HPV16 and HPV18, then direct referral colposcopy (without cytology) is possible [91]. The same sample used for primary testing is recommended to be subsequently used for triage testing in order to reduce the risk of follow-up loss and maximize the efficiency of resources [47,55,91,99]. Furthermore, primary HPV testing improves cytology screening by eliminating HPV-negative ASC-US cases, which constitute a considerable portion of borderline cytology and pose essentially no elevated risk for underlying CIN2/3 or cancer [77,83,91]. Moreover, there is evidence that the predictive value of cytology readouts increases if the cytologist is aware of HPV status of the sample [88]. 

To increase the specificity and improve the detection of precancerous lesions, other techniques, in addition to cytology, that could potentially be used for the triaging of women after a positive HPV primary test result are: partial HPV genotyping (HPV16/18 or extended), p16-Ki67 immunostaining, HPV E6/E7 mRNA detection, and cellular and viral methylation assays. However, at this time, there are insufficient data to favor such methods over cytology for triaging in Europe. The use of partial HPV genotyping triage is based on the fact that there is substantial variation in risk depending on HPV type, but it is still a matter of debate as to which HPV types other than HPV16 (HPV-18, HPV-31, HPV-33, HPV-45) it is worth implementing a routine risk-stratification algorithm [100]. The p16/ki67 dual stain and HPV mRNA testing, have the potential to enable a more accurate distinction between transient HPV infections and those that will potentially progress to precancerous lesions/cancer. The p16/ki67 dual stain has been described as a credible tool that compares favorably to cytology, but both the p16/ki67 dual stain and HPV mRNA testing will need to become more cost-effective in order to compete with cytology. Methylation is in a similar predicament: it is still in the early stages but is displaying great potential as an accurate and promising molecular risk-stratification marker. The objectivity that this method offers, the consistency, and the high throughput potential will make methylation a strong candidate triaging method even if its performance is equivalent to that of cytology [55,101,102]. 

#### 4.3.2. Management of Women after a Positive HPV Primary Test Result and Negative Cytology Triage Results

**Figure 1:**
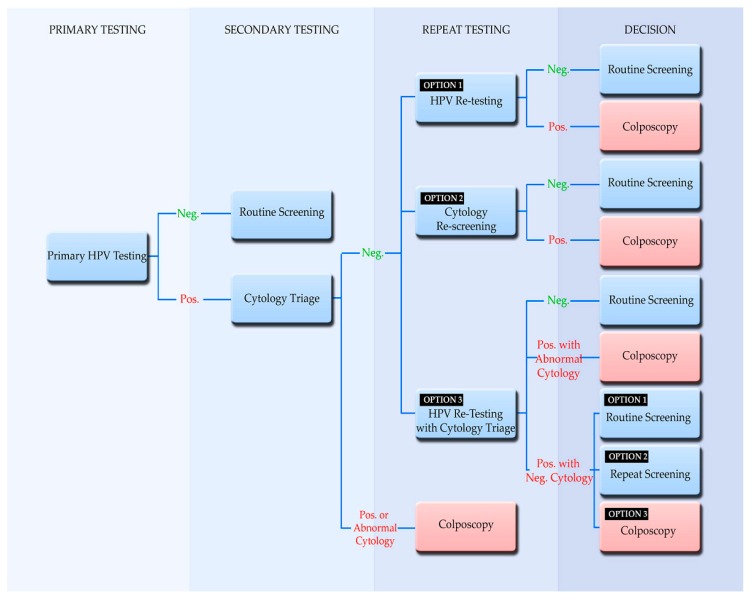
Management algorithm in primary HPV screening. Abnormal cytology refers to a borderline or more severe cytological result. This algorithm was developed based on “The supplements of the second edition of the European Guidelines for Quality Assurance in Cervical Cancer Screening of 2015” [55].

HPV-positive, cytology triage-negative women are recommended to undergo a different path than women with triage-positive cytology and/or borderline cytological results. Cytology triage-negative women who are infected by hrHPV, are still at risk for persistent infection and thus, require repeat testing at shorter intervals than HPV-negative women [83]. The open issue is how to select the most appropriate follow-up test and intervals for repeat testing. The European Guidelines report that at present the evidence available is not sufficient to definitively recommend a single approach for all settings [55] and as such provide three strategies for repeat testing (Figure 1). It is important to note that HPV retesting may be performed after at least 12 months, while cytology retesting can be performed after 6–12 months [99,103,104]. As shown in Figure 1, the European Guidelines recommend that if HPV retesting is performed, a woman with a negative repeat HPV test is recommended to return to routine screening, while a woman with a positive result should be referred to colposcopy. If cytology retesting is performed, a woman with abnormal cytology should be referred for colposcopy, whereas a woman with negative cytology could return to routine screening. If HPV testing with cytology triage in repeat testing is performed it can be managed as follows: A woman with a negative HPV result can return to routine testing. However, a woman with a positive HPV result and abnormal cytology should be referred immediately for colposcopy. A woman with a positive HPV result and negative cytology can be referred to undergo repeat testing after 12 months, for colposcopy, or return to routine screening [99,105,106]. A recent study, however, discourages the use of HPV repeat testing since women who test repeatedly HPV positive and cytology negative still have an increased risk for CIN2+ even after a repeat an HPV-negative test [88,107]. This finding also indicates the lack of sufficient evidence regarding repeat testing, and thus, prior to the implementation of HPV based screening in repeat screening, the decision makers of each program have to consider the prevalence of HPV types in the target population as well as the quality of cytology in that region [55].

#### 4.3.3. Post-Treatment Follow-up

Following the referral of a patient for colposcopy, the identification of high-grade cervical lesions may be diagnosed after biopsy (in approximately a quarter of referred women), followed by surgical treatment. Various treatments of high-grade cervical lesions are available, including cryotherapy, laser, loop electrosurgical excision procedure (LEEP) or large loop excision of the transformation zone (LLETZ) and cone biopsy, which are all characterized by an overall high success rate. However, treatment may fail with regard to residual or recurrent precancer, with 5–15% of treated women being diagnosed again with CIN2+ and therefore requiring additional therapy. Indeed, women once diagnosed with high-grade lesions are characterized by an increased lifetime risk of developing cervical cancer [47,108]. Therefore, the increased risk of cancer highlights the importance of close post-treatment monitoring (follow-up testing) with the objective of early identification of residual/recurrent disease [108,109,110]. For many years, the Pap test has been the most widely employed follow-up test, despite having relatively low sensitivity in this setting. Since 2008, the European Guidelines have recommended the performance of cytology 6, 12, and 24 months after CIN2+ treatment as main follow-up test [111,112,113]. Nonetheless, there is growing evidence for the use of HPV testing in post-treatment monitoring, either alone or as cotesting. Importantly, cotesting achieves only marginally higher sensitivity than cytology or HPV testing alone, implying that HPV testing can be safely used without cytology [109,113,114]. A study analyzing pooled data from 33 published studies argues in favor of follow-up hrHPV testing by noting that it had higher sensitivity for underlying CIN2+ and comparable sensitivity to that of cytology. In the same study it was also stated that women with positive surgical margins may benefit more from hrHPV testing due to very high PPV and NPV [109]. Nevertheless, large-scale RCTs are required to establish the best follow-up algorithms after treatment of high-grade lesions [109].

## 5. The Current Landscape of Cervical Cancer Screening in the E.U. and Some E.U.-Associated Countries

### 5.1. The Implementation Status of Organized Population-Based Programs for Cervical Cancer Screening

Considering the pros and cons of all available cervical cancer screening tests, and despite the existence of evidence-based recommendations, it is clear that there is no “one size fits all” model for cervical cancer screening. There are various factors affecting the implementation of a screening program: the amount of healthcare funds available in each region/country, the preexisting medical and economic infrastructure, and the risk perception and tolerance of the society [87]. Table 1, which presents the data of each country regarding their cervical cancer screening programs, collected through meticulous bibliographical search, shows clearly that there is significant variation in the way members of the E.U., as well as some E.U. associated countries, address the matter of cervical cancer screening (Table 1). Thus, the most recent official survey of implementation status of cervical cancer screening in the E.U. showed that although substantial improvement in screening implementation was documented in last decade and that a total of 22 member states were implementing, piloting, or planning the population-based cervical cancer screening program in 2016, the roll-out of the screening programs was completed in only nine out of 28 member states: Denmark, Estonia, Finland, Latvia, Poland, Slovenia, Sweden, The Netherlands, and The United Kingdom [115], along with one E.U. associated country, Norway [116]. There are countries among them that do not yet have organized population-based programs, namely, Austria, Bulgaria, Cyprus, Germany, Greece, Luxembourg, Spain, Israel, and Switzerland. However, even though these countries lack the abovementioned screening program, some of them have in place programs with certain elements of organized programs, mostly as a result of recommendations issued by the country’s government and/or national gynecological/medical societies. In Austria, for example, a nationwide opportunistic program was created in 1970 to screen for cervical cancer. The program has remained opportunistic and is loosely structured by recommendations from Austrian medical societies, and the expenses are covered by health insurance [117,118,119,120]. In Israel, screening is recommended and fully covered by the National Health Insurance Law, and furthermore, the Israeli Gynecological Society recommends the extension of screening ages from 35–54 to 25–65 [121,122]. A similar situation is noted in Switzerland, where recommendations are offered by the Swiss Gynecological Society, and Pap testing is covered by health insurance [123].

**Figure 2:**
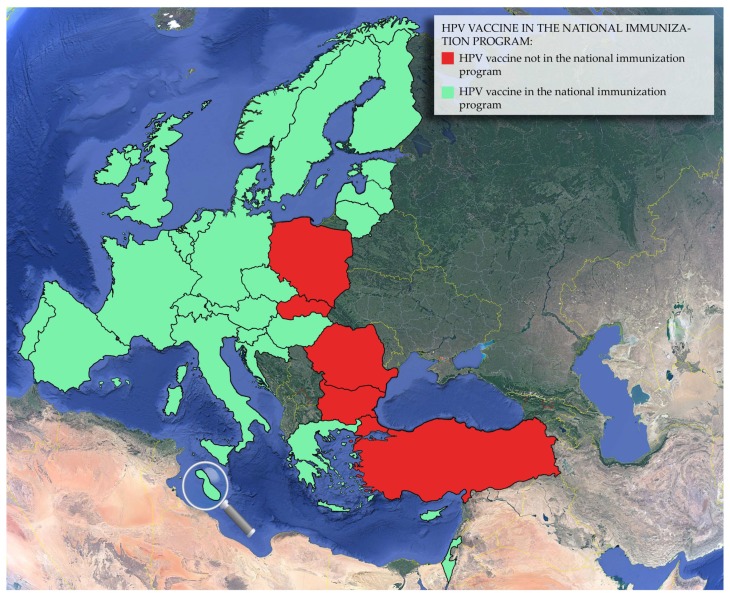
The implementation status of HPV vaccination in E.U. member states and some E.U. associated countries as of 15 May 2018, based on the World Health Organization (WHO) “Vaccine in National Immunization Program Update”. Source: http://www.who.int/immunization/monitoring_surveillance/data/en/; assessed for the last time on 16 July 2018 [131]. The magnifying glass serves to enlarge the island of Malta.

Some of these countries which are still lacking organized national screening programs have made attempts to implement national and/or regional screening programs (Table 1). In 2009, Bulgaria initiated the “Stop and Get Checked” cancer screening program, which ended in 2014 with no scaling up [115,322]. In Cyprus, the Ministry of Health, the Department of Medical and Public Health Services, assigned a temporary committee in 2008 with the intention of implementing a national screening program for cervical cancer in 2009 [118]; however, the program was not realized, and screening is currently opportunistic. Nonetheless, a regional pilot screening program in Cyprus, organized by a private organization of women in cooperation with governmental health services as well as the support of the Ministry of Health, was initiated in 2012 and is still in effect [152]. Similarly, in Greece, a number of regional cervical cancer screening programs have been reported, and there have also been efforts to establish a national organized population-based screening program for cervical cancer. These efforts have not been fruitful yet, reportedly due to the financial crisis [206]. In Luxembourg, a national cervical cancer screening program was initiated in 1962, and it is currently opportunistic, run by a single national cytology laboratory [234,236]. In Spain, screening at a national level is opportunistic, and there are variations in screening recommendations in different regions. In addition, some regions have their own population-based programs. Several scientific Spanish societies recommend the implementation of an organized screening program with HPV primary screening [92,269,278]. Germany, however, with the passing of the Cancer Screening and Registration Law of 2013, has planned for an organized population-based cervical cancer screening program, which was reported to be scheduled for implementation by 2018 [197]. In France, despite the existence of organized population-based programs, the country has been primarily characterized by opportunistic screening. National guidelines were published in 2010 for the initiation of a population-based cervical cancer screening program, and they are expected to be implemented nationwide in 2018 [185]. In Lithuania, the program is organized but still has some opportunistic qualities, since the general practitioners (GPs) are the ones instructing patients to attend cervical cancer screening instead of the process being governed by an organized call-recall system and the invitations being sent out by mail [232]. In Turkey, there are both organized and opportunistic programs, but the opportunistic approach is employed to a higher degree. An organized screening program implemented in 2004 was characterized by low coverage and redesigned in 2014 to include primary HPV testing, with the additional implementation of HPV vaccination being debated as well [312,315,316,317,318]. Besides Turkey, other countries covered in this review that have yet to implement an HPV vaccination program are Bulgaria, Poland, Romania and Slovakia, indicating that HPV vaccination programs have been adopted by the majority of members of the E.U. and E.U. associated countries. As presented in Table 1 and depicted in Figure 2, out of the 32 countries covered in this review, only five countries do not have a national HPV vaccination program running [131].

### 5.2. The Implementation Status of Primary HPV Testing

**Figure 3:**
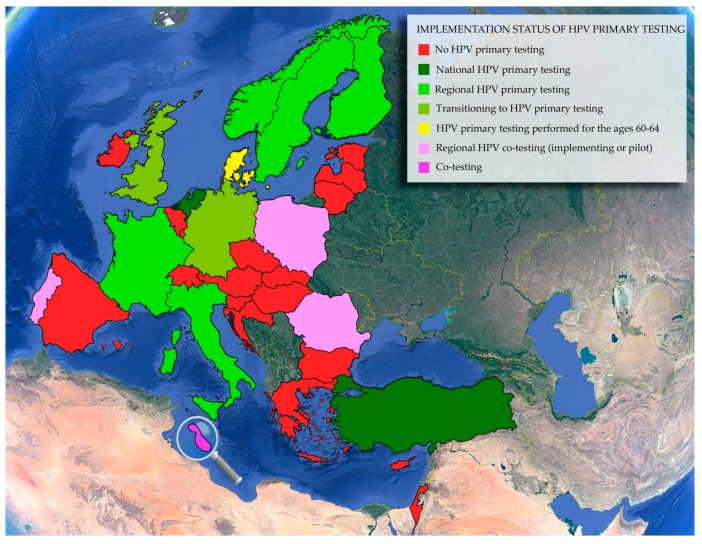
The implementation status of primary HPV testing in E.U. member states and some E.U. associated countries. The magnifying glass serves to enlarge the island of Malta. It is important to state that this is a rapidly changing field and that the status of implementation could not be confirmed for all countries from two independent sources.

As it can be observed from the data in Table 1, which provides the implementation status of HPV primary testing in each country, there is a recent movement towards HPV-based primary screening, which has been embraced by some countries and is currently being strongly considered by others (Figure 3). Finland, Germany, Italy, The Netherlands, Sweden, The United Kingdom, Norway, and Turkey are all either in the process of implementing HPV primary screening on a regional or national level or have done so recently. Distinctions should be noted for Norway, where a regional pilot program for HPV primary testing is underway, and Finland, where HPV primary screening is implemented by some municipalities [175,184]. In France, primary HPV testing has been studied in regional pilot programs [6,115,186]. Romania is currently using cotesting in some regions and reportedly the strategy is to change to HPV primary screening during the 2017–2020 National Cancer Control Plan [115,257]. Cotesting was also employed for a pilot study in two regions of Poland [248] and for some regions in Portugal [251]. Moreover, cotesting is also being performed in a pilot population-based program that is still ongoing in Malta [115]. Other countries, such as Denmark, which performs HPV testing for women in the age range of 60–64 [169,170] and Belgium [138], are still evaluating HPV primary screening for implementation in their national programs.

### 5.3. The Importance of Coverage and Acceptance of Cervical Cancer Screening Programs

Despite all the efforts to implement screening programs, their success depends primarily on sufficient population coverage. Unfortunately, many countries report suboptimal participation in screening programs [210,211,229,232,315]. In an effort to increase coverage, in addition to educational campaigns and invitation reminders, many countries are also exploring or implementing self-sampling for nonparticipants [183,204,293,307,309,323,324]. This testing strategy is also mentioned in the European Guidelines; however, they recommend that successful self-sampling pilot projects precede implementation. Furthermore, it is important to emphasize that self-sampling should be performed for HPV testing and not cytology [55,325]. HPV self-sampling has been reported to have similar sensitivity and specificity as testing performed on samples taken by trained professionals. However, European Guidelines do not recommend self-sampling for all women, since, although they performed similarly, the results of self-collected samples are less accurate than those of samples collected by clinicians [55]. The acceptability of self-sampling for HPV testing was shown in an RCT, where 99% of the samples returned were adequate for analysis, indicating that self-sampling can be a valid alternative for nonparticipants [326].

**Figure 4:**
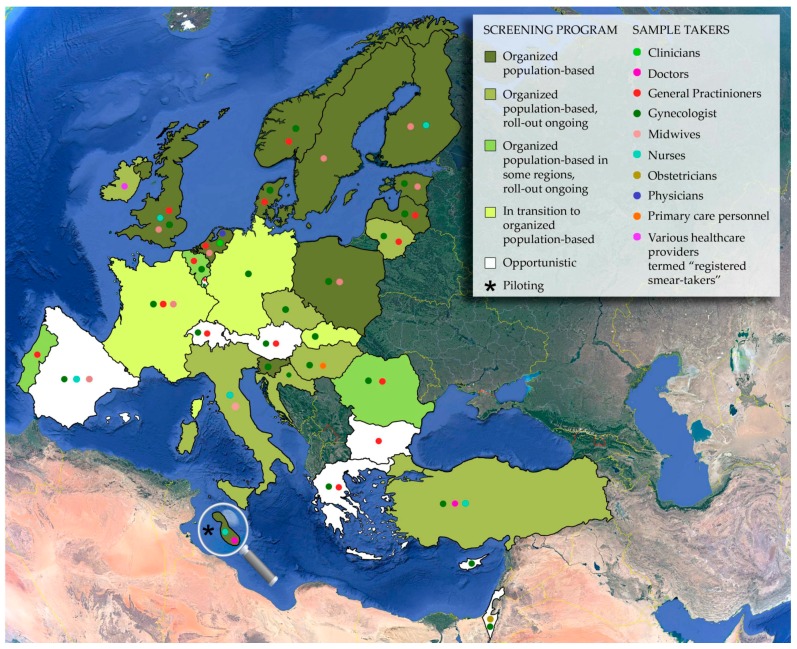
Health care providers that act as sample takers in cervical cancer screening programs in E.U. member states and some E.U. associated countries. The magnifying glass serves to enlarge the island of Malta. It is important to state that this is a rapidly changing field and that the status of implementation could not be confirmed from two independent sources and that this is a rapidly changing field. This figure was designed based on information available in Table 1 and Basu et al., 2017 [115].

Low coverage is directly affected by the targeted population, and accordingly, there have been numerous studies in various countries evaluating the awareness, perception, and knowledge of the population in regard to HPV, cervical cancer screening programs and vaccination programs [141,172,205,209,219,247,251,255,258,283,320,321]. These studies also highlight the importance of health care providers, general practitioners and gynecologists, both in opportunistic screening and in organized programs [128,207,327]. As indicated in Table 1 and illustrated in Figure 4, GPs and gynecologists tend to be the primary figures in opportunistic screening, performing the examinations and collecting the specimens, while in organized settings, the specimen can be can be collected by a variety of medically qualified individuals, such as nurses and midwives. These factors emphasize the importance of all affected parties in the movement towards organized population-based HPV primary screening. All parties must work together in order to achieve success, whether an already existing cytology-based organized program is upgraded to HPV-based program or a new organized program is implemented in a country previously performing opportunistic screening only.

## 6. Conclusions

Cervical cancer is an important health care problem in many parts of the world as well as in the E.U. It is a disease with a clearly defined natural history caused by essentially one etiological agent, and with long clinical latency. These characteristics of the disease enabled the development of acceptable and valid testing, such as the Pap test that was invented in the 1940s, to identify the precursor lesions, which can be treated in a safe, effective and acceptable way. This subsequently led to the establishment of routine cervical cancer screening in the 1960s. Primary prevention of cervical cancer was implemented more recently with the release of the first prophylactic HPV vaccine in 2006. Currently, the European Guidelines recommend organized population-based screening with primary HPV testing. However, this paradigm shift requires either the reformation of currently existing cytology-based organized programs or the implementation of new programs for countries still relying on opportunistic screening, which also mainly use cytology as a screening tool. The existing cytology-based screening programs are in many instances inefficient and costly because of the subjective nature of cytology, threatening to strain the public health budget of many countries, an effect that is expected to be exacerbated further as population HPV vaccination coverage increases [84]. We are all fully aware that the implementation of functioning HPV-based organized cervical screening programs with accessible and effective treatment of precancerous lesions, coupled with universal gender-neutral HPV vaccination, is challenging for some of the E.U. member states, however, this is certainly the only way forward. When adequately combined, these two promising prevention options have the potential to dramatically reduce cervical cancer incidence and mortality.

## Figures and Tables

**Table 1:** Data regarding cervical cancer screening and human papillomavirus (HPV) vaccination programs of European Union (E.U.) members and some E.U. associated countries.

Countries	Presence and Type of Cervical Screening Program (Year of Initiation)	Screening Ages (Years)	Screening Interval (Years)	Primary Screening Test Used	Sample Taker	Use of HPV Testing	HPV Vaccine in the National Immunization Program (Year of Initiation)	References
**E.U. Member Countries**								
**Austria**	Opportunistic	18+ or 2 years after sexual onset	1	CC	GYN, GPs	HPV testing funded in certain cases	2014	[117,120,124,125,126,127,128,129,130,131]
**Belgium ^1^**	Organized population-based, in some regions, roll-out ongoing, 2013	25–64	3	CC & LBC	GYN, GPs	HPV triage testing	2010: Flemish community, 2011: French community	[131,132,133,134,135,136,137,138,139,140,141,142,143]
**Bulgaria**	Opportunistic	N/A	N/A	N/A	GPs	N/A	-	[115,131,144,145,146,147,148]
**Croatia**	Organized population-based, roll-out ongoing, 2012	25–64	3	CC	GYN	HPV triage testing, test for cure	2016	[115,131,149,150]
**Cyprus ^2^**	Opportunistic	N/A	N/A	N/A	GYN	N/A	2016	[131,151,152,153,154]
**Czech Republic**	Organized population-based, roll-out ongoing, 2008	15+	1	CC	GYN	HPV triage testing	2012	[115,131,155,156,157,158,159,160]
**Denmark**	Organized population-based, 2006	23–64	3 (ages: 23–59); 5 (ages: 60–64)	LBC (ages: 23–59); HPV test (ages: 60–64)	GYN, GPs	HPV primary testing performed for the ages 60–64, HPV triage testing, test for cure	2009	[6,131,161,162,163,164,165,166,167,168,169,170,171]
**Estonia**	Organized population-based, 2006	30–59	5	CC	GYN, Midwives	Not in use	2018	[6,131,172,173,174]
**Finland ^3^**	Organized population-based, 1963	25/30–60/65	5	CC, HPV test	Midwives, Nurses	HPV primary testing in some regions, HPV triage testing	2013	[131,175,176,177,178,179,180,181,182,183,184]
**France**	Transitioning to organized population-based planned for 2018, 1991	25–64	3 (CC), 5 (HPV test)	CC & LBC (ages: 25–64); HPV test (ages: 30–64)	GYN, GPs, Midwives	HPV primary testing in regional pilot projects, HPV triage testing	2007	[6,115,131,142,160,185,186,187,188,189,190,191,192]
**Germany**	Transitioning to organized population-based planned for 2018, 1971	20+	1	CC	GYN	HPV primary testing in implementation, HPV triage testing	2007	[64,115,131,160,193,194,195,196,197,198,199,200,201]
**Greece ^4^**	Opportunistic	Sexual onset	1	CC	GYN, GPs	Not in use	2008	[128,131,202,203,204,205,206,207]
**Hungary**	Organized population-based, roll-out ongoing, 2003	25–65	3	CC	GYN, Primary care personnel	Not in use	2014	[6,115,131,208,209,210,211,212,213,214]
**Ireland**	Organized population-based, roll-out ongoing, 2008	25–60	3 (ages: 25–44); 5 (ages: 45–60)	LBC	Various healthcare providers termed registered smear-takers	HPV triage testing, test for cure	2010	[6,115,131,215,216,217,218,219]
**Italy ^3^**	Organized population-based, roll-out ongoing, 1989	25–64	3 (ages: 25–30/35); 5 (ages: 30/35–64)	CC & LBC (ages: 25–30/35); HPV test (ages: 30/34–64)	Midwives, Nurses	HPV primary testing in some regions, HPV triage testing, test for cure	2008	[6,115,118,131,220,221,222,223,224,225,226,227]
**Latvia**	Organized population-based, 2009	25–69	3	CC	GYN, GPs	Not in use	2010	[6,131,228,229,230,231]
**Lithuania**	Organized population-based, roll-out ongoing, 2004	25–59	3	CC	GYN, GPs	Not in use	2016	[6,67,115,131,232,233]
**Luxembourg**	Opportunistic	15+	1	LBC	GYN, GPs	HPV triage testing	2008	[131,234,235,236,237,238,239]
**Malta**	Organized population-based, (Piloting), 2015	25–35	3	CC & HPV test	Doctors, Nurses	Cotesting	2012	[67,115,131,159,160,240]
**Netherlands**	Organized population-based, 1970	30–64	5	HPV test	GPs, Physicians, Clinicians, Midwives	HPV primary testing, HPV triage testing	2010	[87,115,131,241,242,243,244,245,246]
**Poland**	Organized population-based, 2006	25–59	3	CC (ages: 25–59); CC & HPV test (ages: 30–59)	GYN, Midwives	Regional pilot for Cotesting, HPV triage testing	-	[6,115,131,160,247,248,249,250]
**Portugal ^3^**	Organized population-based, in some regions, roll-out ongoing, 1990	25–59	3	CC & LBC	GPs	Cotesting in some regions	2008	[115,131,251,252,253,254]
**Romania ^5^**	Organized population-based, in some regions, roll-out ongoing, 2012	25–64	5	CC & HPV test	GYN, GPs	Cotesting in some regions	-	[115,131,255,256,257,258,259,260]
**Slovakia**	Transitioning to organized population-based, 2008	23–64	Yearly x 2; then 3 yearly	CC	GYN	HPV triage testing	-	[75,115,131,147,159,261,262,263,264]
**Slovenia**	Organized population-based, 2003	20–64	Yearly x 2; then 3 yearly	CC	GYN	HPV triage testing, test of cure	2009	[6,115,131,147,160,265,266,267,268]
**Spain ^3^**	Opportunistic	25–65	3	CC	GYN, Nurses, Midwives	HPV triage testing in some regions	2007	[92,115,118,131,160,269,270,271,272,273,274,275,276,277,278]
**Sweden**	Organized population-based, 1967	23–60	3 (ages: 23–50); 5 (ages: 51–60)	HPV test replacing CC & LBC	Midwives	HPV primary testing in some regions HPV triage testing, test for cure	2012	[114,115,131,142,279,280,281,282,283,284,285,286]
**United Kingdom**	Organized population-based, 1988	25–64	3 (ages: 25–49); 5 (ages: 50–64)	HPV test replacing LBC	GYN, GPs, Nurses, Midwives	HPV primary testing in implementation, HPV triage testing, test for cure	2008	[115,128,131,287,288,289,290,291,292,293]
**E.U. Associated Countries**								
**Norway**	Organized population-based, 1995	25–69	3	CC & LBC, HPV test	GYN, GPs	Regional pilot for HPV primary testing, HPV triage testing	2009	[131,294,295,296,297,298,299]
**Israel**	Opportunistic	35–54	3	LBC	GYN, Obstetricians	Not in use	2013	[121,122,131,300,301,302,303,304]
**Switzerland**	Opportunistic	Sexual onset/21–70	2 (ages: Sexual onset/21–29); 3 (ages: 30–70)	CC & LBC	GYN, GPs	HPV triage testing	2008	[69,123,131,305,306,307,308,309,310,311]
**Turkey**	Organized population-based, roll-out ongoing, 2004	30–65	5	HPV test, CC & LBC,	GYN, Doctors, Nurses	HPV primary testing	-	[131,312,313,314,315,316,317,318,319,320,321]

Conventional cytology; LBC = liquid-based cytology; GP = general practitioner; N/A = not available, GYN = gynecologists; “-” = HPV vaccine not in the national immunization program. ^1^ In Belgium an organized population-based program is in place only in the Flemish region [138]. ^2^ In Cyprus a regional pilot screening program was initiated in 2012, which is still in effect [152]. ^3^ In Finland, Italy, Portugal and Spain there is variation depending on the region. There are some regions in Spain that have population-based programs [92,175,184,220,251,269,278,312]. ^4^ In Greece, there are some regional cervical cancer screening programs that have been reported [206]. ^5^ In Romania an HPV vaccination program had started in 2008 but it was discontinued due to low uptake [256,257,259].

## References

[1] Ferlay J., Soerjomataram I., Dikshit R., Eser S., Mathers C., Rebelo M., Parkin D.M., Forman D., Bray F. (2015). Cancer incidence and mortality worldwide: Sources, methods and major patterns in GLOBOCAN 2012. Int. J. Cancer.

[2] Bruni L., Albero G., Serrano B., Mena M., Gómez D., Muñoz J., Bosch F.X., de Sanjosé S., ICO/IARC Information Centre on HPV and Cancer (HPV Information Centre) Human Papillomavirus and Related Diseases Report in EUROPE. http://www.hpvcentre.net/statistics/reports/XEX.pdf.

[3] Shingleton H.M., Patrick R.L., Johnston W.W., Smith R.A. (1995). The current status of the Papanicolaou smear. CA Cancer J. Clin..

[4] Petry K.U. (2014). HPV and cervical cancer. Scand. J. Clin. Lab. Investig..

[5] Koh W.-J., Greer B.E., Abu-Rustum N.R., Apte S.M., Campos S.M., Cho K.R., Chu C., Cohn D., Crispens M.A., Dorigo O. (2015). Cervical Cancer, Version 2.2015. J. Natl. Compr. Cancer Netw..

[6] Elfström K.M., Arnheim-Dahlström L., von Karsa L., Dillner J. (2015). Cervical cancer screening in Europe: Quality assurance and organisation of programmes. Eur. J. Cancer.

[7] Ferlay J., Steliarova-Foucher E., Lortet-Tieulent J., Rosso S., Coebergh J.W.W., Comber H., Forman D., Bray F. (2013). Cancer incidence and mortality patterns in Europe: Estimates for 40 countries in 2012. Eur. J. Cancer.

[8] Bray F., Ren J.-S., Masuyer E., Ferlay J. (2013). Global estimates of cancer prevalence for 27 sites in the adult population in 2008. Int. J. Cancer.

[9] Bray F., Loos A.H., McCarron P., Weiderpass E., Arbyn M., Møller H., Hakama M., Parkin D.M. (2005). Trends in Cervical Squamous Cell Carcinoma Incidence in 13 European Countries: Changing Risk and the Effects of Screening. Cancer Epidemiol. Biomarkers Prev..

[10] Dürst M., Gissmann L., Ikenberg H., zur Hausen H. (1983). A papillomavirus DNA from a cervical carcinoma and its prevalence in cancer biopsy samples from different geographic regions. Proc. Natl. Acad. Sci. USA.

[11] Cox J.T. (2009). History of the use of HPV testing in cervical screening and in the management of abnormal cervical screening results. J. Clin. Virol..

[12] Walboomers J.M.M., Jacobs M.V., Manos M.M., Bosch F.X., Kummer J.A., Shah K.V., Snijders P.J.F., Peto J., Meijer C.J.L.M., Muñoz N. (1999). Human papillomavirus is a necessary cause of invasive cervical cancer worldwide. J. Pathol..

[13] Clifford G.M., Smith J.S., Plummer M., Muñoz N., Franceschi S. (2003). Human papillomavirus types in invasive cervical cancer worldwide: A meta-analysis. Br. J. Cancer.

[14] Mühr L.S.A., Eklund C., Dillner J. (2018). Towards quality and order in human papillomavirus research. Virology.

[15] Lyu Z., Feng X., Li N., Zhao W., Wei L., Chen Y., Yang W., Ma H., Yao B., Zhang K. (2017). Human papillomavirus in semen and the risk for male infertility: A systematic review and meta-analysis. BMC Infect. Dis..

[16] Chabeda A., Yanez R.J.R., Lamprecht R., Meyers A.E., Rybicki E.P., Hitzeroth I.I. (2018). Therapeutic vaccines for high-risk HPV-associated diseases. Papillomavirus Res..

[17] Tommasino M. (2014). The human papillomavirus family and its role in carcinogenesis. Semin. Cancer Biol..

[18] Moscicki A.-B., Schiffman M., Kjaer S., Villa L.L. (2006). Chapter 5: Updating the natural history of HPV and anogenital cancer. Vaccine.

[19] Berman T.A., Schiller J.T. (2017). Human papillomavirus in cervical cancer and oropharyngeal cancer: One cause, two diseases. Cancer.

[20] Ermel A., Shew M.L., Imburgia T.M., Brown M., Qadadri B., Tong Y., Brown D.R. (2018). Redetection of human papillomavirus type 16 infections of the cervix in mid-adult life. Papillomavirus Res..

[21] Ostör A.G. (1993). Natural history of cervical intraepithelial neoplasia: A critical review. Int. J. Gynecol. Pathol..

[22] De Villiers E.-M. (2013). Cross-roads in the classification of papillomaviruses. Virology.

[23] Xi L.F., Schiffman M., Koutsky L.A., Hughes J.P., Hulbert A., Shen Z., Galloway D.A., Kiviat N.B. (2016). Variant-specific persistence of infections with human papillomavirus Types 31, 33, 45, 56 and 58 and risk of cervical intraepithelial neoplasia. Int. J. Cancer.

[24] Halec G., Alemany L., Lloveras B., Schmitt M., Alejo M., Bosch F.X., Tous S., Klaustermeier J.E., Guimerà N., Grabe N. (2014). Pathogenic role of the eight probably/possibly carcinogenic HPV types 26, 53, 66, 67, 68, 70, 73 and 82 in cervical cancer. J. Pathol..

[25] Muñoz N., Bosch F.X., de Sanjosé S., Herrero R., Castellsagué X., Shah K.V., Snijders P.J.F., Meijer C.J.L.M. (2003). Epidemiologic Classification of Human Papillomavirus Types Associated with Cervical Cancer. N. Engl. J. Med..

[26] Smith J.S., Lindsay L., Hoots B., Keys J., Franceschi S., Winer R., Clifford G.M. (2007). Human papillomavirus type distribution in invasive cervical cancer and high-grade cervical lesions: A meta-analysis update. Int. J. Cancer.

[27] Breitenecker G. (2009). Zervixkarzinom-Screening. Der Pathologe.

[28] Diamantis A., Magiorkinis E. (2014). Pioneers of exfoliative cytology in the 19th century: The predecessors of George Papanicolaou. Cytopathology.

[29] Tan S.Y., Tatsumura Y. (2015). George Papanicolaou (1883–1962): Discoverer of the Pap smear. Singap. Med. J..

[30] Chantziantoniou N., Donnelly A.D., Mukherjee M., Boon M.E., Austin R.M. (2017). Inception and Development of the Papanicolaou Stain Method. Acta Cytol..

[31] Papanicolaou G.N., Traut H.F. (1941). The Diagnostic Value of Vaginal Smears in Carcinoma of the Uterus. Am. J. Obstet. Gynecol..

[32] Dighe S.B., Ajit D., Pathuthara S., Chinoy R. (2006). Papanicolaou Stain. Acta Cytol..

[33] Siebers A.G., Klinkhamer P.J., Grefte J.M., Massuger L.F., Vedder J.E., Beijers-Broos A., Bulten J., Arbyn M. (2009). Comparison of liquid-based cytology with conventional cytology for detection of cervical cancer precursors: A randomized controlled trial. JAMA.

[34] Gibb R.K., Martens M.G. (2011). The Impact of Liquid-Based Cytology in Decreasing the Incidence of Cervical Cancer. Rev. Obstet. Gynecol..

[35] Prasaad P.R. (2017). Short-duration Papanicolaou stain (SPS)—An alternative to conventional Papanicolaou stain in routine cytopathology?. Comp. Clin. Pathol..

[36] Yang G.C., Alvarez I.I. (1995). Ultrafast Papanicolaou stain. An alternative preparation for fine needle aspiration cytology. Acta Cytol..

[37] Gill G.W. (2006). Enviro-Pap: An Environmentally Friendly, Economical, and Effective Pap Stain. Lab. Med..

[38] Thakur M., Guttikonda V.R. (2017). Modified ultrafast Papanicolaou staining technique: A comparative study. J. Cytol..

[39] Izhar S., Kaur R., Masih K. (2014). Efficacy of rapid, economical, acetic acid, Papanicolaou stain in cervical smears as an alternative to conventional Papanicolaou stain. J. Cytol./Indian Acad. Cytol..

[40] Lyng F.M., Traynor D., Ramos I.R.M., Bonnier F., Byrne H.J. (2015). Raman spectroscopy for screening and diagnosis of cervical cancer. Anal. Bioanal. Chem..

[41] Strander B., Andersson-Ellström A., Milsom I., Rådberg T., Ryd W. (2007). Liquid-based cytology versus conventional Papanicolaou smear in an organized screening program. Cancer Cytopathol..

[42] Sakamoto H., Takenaka M., Ushimaru K., Tanaka T. (2012). Use of Liquid-Based Cytology (LBC) and Cell Blocks from Cell Remnants for Cytologic, Immunohistochemical, and Immunocytochemical Diagnosis of Malignancy. Open J. Pathol..

[43] Moseley R.P., Paget S. (2002). Liquid-based cytology: Is this the way forward for cervical screening?. Cytopathology.

[44] Davey E., Barratt A., Irwig L., Chan S.F., Macaskill P., Mannes P., Saville A.M. (2006). Effect of study design and quality on unsatisfactory rates, cytology classifications, and accuracy in liquid-based versus conventional cervical cytology: A systematic review. Lancet.

[45] Jeong H., Hong S.R., Chae S.-W., Jin S.-Y., Yoon H.K., Lee J., Kim E.K., Ha S.T., Kim S.N., Park E.-J. (2017). Comparison of Unsatisfactory Samples from Conventional Smear versus Liquid-Based Cytology in Uterine Cervical Cancer Screening Test. J. Pathol. Transl. Med..

[46] Singh V., Gupta N., Nijhawan R., Srinivasan R., Suri V., Rajwanshi A. (2015). Liquid-based cytology versus conventional cytology for evaluation of cervical Pap smears: Experience from the first 1000 split samples. Indian J. Pathol. Microbiol..

[47] Basu P., Mittal S., Bhadra Vale D., Chami Kharaji Y. (2018). Secondary prevention of cervical cancer. Best Pract. Res. Clin. Obstet. Gynaecol..

[48] Huchko M.J., Sneden J., Zakaras J.M., Smith-McCune K., Sawaya G., Maloba M., Bukusi E.A., Cohen C.R. (2015). A Randomized Trial Comparing the Diagnostic Accuracy of Visual Inspection with Acetic Acid to Visual Inspection with Lugol’s Iodine for Cervical Cancer Screening in HIV-Infected Women. PLoS ONE.

[49] Belinson J., Pretorius R., Zhang W., Wu L., Qiao Y., Elson P. (2001). Cervical cancer screening by simple visual inspection after acetic acid. Obstet. Gynecol..

[50] Soloman D. (1990). The 1988 bethesda system for reporting cervical/vaginal cytologic diagnoses: Developed and approved at the National Cancer Institute workshop in Bethesda, Maryland, December 12–13, 1988. Hum. Pathol..

[51] Nayar R., Wilbur D.C. (2015). The Bethesda System for Reporting Cervical Cytology: Definitions, Criteria, and Explanatory Notes.

[52] Stoler M.H., Ronnett B.M., Joste N.E., Hunt W.C., Cuzick J., Wheeler C.M., New Mexico HPV Pap Registry Steering Committee (2015). The Interpretive Variability of Cervical Biopsies and its Relationship to HPV status. Am. J. Surg. Pathol..

[53] Arbyn M., Ronco G., Anttila A., Meijer C.J.L.M., Poljak M., Ogilvie G., Koliopoulos G., Naucler P., Sankaranarayanan R., Peto J. (2012). Evidence Regarding Human Papillomavirus Testing in Secondary Prevention of Cervical Cancer. Vaccine.

[54] Von Karsa L., Arbyn M., De Vuyst H., Dillner J., Dillner L., Franceschi S., Patnick J., Ronco G., Segnan N., Suonio E. (2015). European guidelines for quality assurance in cervical cancer screening. Summary of the supplements on HPV screening and vaccination. Papillomavirus Res..

[55] Von Karsa L., Arbyn A., De Vuyst H., Dillner J., Dillner L., Franceschi S., Patnick J., Ronco G., Segnan N., Suonio E. (2015). Executive summary. European Guidelines for Quality Assurance in Cervical Cancer Screening.

[56] Poljak M., Kocjan B.J. (2010). Commercially available assays for multiplex detection of alpha human papillomaviruses. Expert Rev. Anti-infect. Ther..

[57] Poljak M., Cuzick J., Kocjan B.J., Iftner T., Dillner J., Arbyn M. (2012). Nucleic Acid Tests for the Detection of Alpha Human Papillomaviruses. Vaccine.

[58] Poljak M., Kocjan B.J., Oštrbenk A., Seme K. (2016). Commercially available molecular tests for human papillomaviruses (HPV): 2015 update. J. Clin. Virol..

[59] Arbyn M., Snijders P.J.F., Meijer C.J.L.M., Berkhof J., Cuschieri K., Kocjan B.J., Poljak M. (2015). Which high-risk HPV assays fulfil criteria for use in primary cervical cancer screening?. Clin. Microbiol. Infect..

[60] Ronco G., Giorgi-Rossi P., Carozzi F., Confortini M., Palma P.D., Del Mistro A., Ghiringhello B., Girlando S., Gillio-Tos A., De Marco L. (2010). Efficacy of human papillomavirus testing for the detection of invasive cervical cancers and cervical intraepithelial neoplasia: A randomised controlled trial. Lancet Oncol..

[61] Meijer C., Berkhof J., Castle P.E., Hesselink A., Franco E.L., Ronco G., Arbyn M., Bosch F.X., Cuzick J., Dillner J. (2009). Guidelines for human papillomavirus DNA test requirements for primary cervical cancer screening in women of 30 years and older. Int. J. Cancer.

[62] Arbyn M., Depuydt C., Benoy I., Bogers J., Cuschieri K., Schmitt M., Pawlita M., Geraets D., Heard I., Gheit T. (2016). VALGENT: A protocol for clinical validation of human papillomavirus assays. J. Clin. Virol..

[63] Muñoz N., Kjaer S.K., Sigurdsson K., Iversen O.-E., Hernandez-Avila M., Wheeler C.M., Perez G., Brown D.R., Koutsky L.A., Tay E.H. (2010). Impact of Human Papillomavirus (HPV)-6/11/16/18 Vaccine on All HPV-Associated Genital Diseases in Young Women. JNCI J. Natl. Cancer Inst..

[64] Schülein S., Taylor K.J., König J., Claus M., Blettner M., Klug S.J. (2016). Factors influencing uptake of HPV vaccination among girls in Germany. BMC Public Health.

[65] Dilley S., Miller K., Huh W. (2018). HPV vaccination. Gynecol. Oncol..

[66] Braaten K.P., Laufer M.R. (2008). Human Papillomavirus (HPV), HPV-Related Disease, and the HPV Vaccine. Rev. Obstet. Gynecol..

[67] Brotherton J.M.L., Bloem P.N. (2017). Population-based HPV vaccination programmes are safe and effective: 2017 update and the impetus for achieving better global coverage. Best Pract. Res. Clin. Obstet. Gynaecol..

[68] Higgins L.M., Dirksing K.N., Ding L., Morrow C.D., Widdice L.A., Kahn J.A. (2016). Adolescents’ intention and self-efficacy to follow Pap testing recommendations after receiving the HPV vaccine. Hum. Vaccines Immunother..

[69] Jacot-Guillarmod M., Pasquier J., Greub G., Bongiovanni M., Achtari C., Sahli R. (2017). Impact of HPV vaccination with Gardasil^®^ in Switzerland. BMC Infect. Dis..

[70] Meites E., Kempe A., Markowitz L.E. (2017). Use of a 2-Dose Schedule for Human Papillomavirus Vaccination—Updated Recommendations of the Advisory Committee on Immunization Practices. Am. J. Transplant..

[71] Iversen O.E., Miranda M.J., Ulied A., Soerdal T., Lazarus E., Chokephaibulkit K., Block S.L., Skrivanek A., Nur Azurah A.G., Fong S.M. (2016). Immunogenicity of the 9-valent hpv vaccine using 2-dose regimens in girls and boys vs a 3-dose regimen in women. JAMA.

[72] Jiang R.T., Wang J.W., Peng S., Huang T.-C., Wang C., Cannella F., Chang Y.-N., Viscidi R.P., Best S.R.A., Hung C.-F. (2017). Spontaneous and Vaccine-Induced Clearance of Mus Musculus Papillomavirus 1 Infection. J. Virol..

[73] Romanowski B., Schwarz T.F., Ferguson L., Peters K., Dionne M., Behre U., Schulze K., Hillemanns P., Suryakiran P., Thomas F. (2016). Sustained immunogenicity of the HPV-16/18 AS04-adjuvanted vaccine administered as a two-dose schedule in adolescent girls: Five-year clinical data and modeling predictions from a randomized study. Hum. Vaccines Immunother..

[74] Stanley M. (2014). HPV vaccination in boys and men. Hum. Vaccines Immunother..

[75] Audisio R.A., Icardi G., Isidori A.M., Liverani C.A., Lombardi A., Mariani L., Mennini F.S., Mitchell D.A., Peracino A., Pecorelli S. (2016). Public health value of universal HPV vaccination. Crit. Rev. Oncol./Hematol..

[76] Bosch F.X., Robles C., Díaz M., Arbyn M., Baussano I., Clavel C., Ronco G., Dillner J., Lehtinen M., Petry K.-U. (2015). HPV-FASTER: Broadening the scope for prevention of HPV-related cancer. Nat. Rev. Clin. Oncol..

[77] Nygård M. (2011). Screening for cervical cancer: When theory meets reality. BMC Cancer.

[78] Wilson J.M.G., Jungner G., World Health Organization (1968). Principles and Practice of Screening for Disease.

[79] Coleman D., Day N., Douglas G., Farmery E., Lynge E., Philip J., Segnan N. (1993). European Guidelines for Quality Assurance in Cervical Cancer Screening. Europe against cancer programme. Eur. J. Cancer.

[80] Arbyn M., Anttila A., Jordan J., Ronco G., Schenck U., Segnan N., Wiener H.G., Herbert A., Daniel J., von Karsa L. (2008). European Guidelines for Quality Assurance in Cervical Cancer Screening.

[81] Arbyn M., Anttila A., Jordan J., Ronco G., Schenck U., Segnan N., Wiener H., Herbert A., von Karsa L. (2010). European Guidelines for Quality Assurance in Cervical Cancer Screening. Second Edition—Summary Document. Ann. Oncol..

[82] Hanselaar A.G.J.M. (2002). Criteria for Organized Cervical Screening Programs. Acta Cytol..

[83] Wentzensen N., Schiffman M., Palmer T., Arbyn M. (2016). Triage of HPV positive women in cervical cancer screening. J. Clin. Virol..

[84] Franco E.L., Cuzick J., Hildesheim A., de Sanjosé S. (2006). Chapter 20: Issues in planning cervical cancer screening in the era of HPV vaccination. Vaccine.

[85] Toliman P.J., Kaldor J.M., Tabrizi S.N., Vallely A.J. (2018). Innovative approaches to cervical cancer screening in low- and middle-income countries. Climacteric.

[86] Isidean S.D., Mayrand M.H., Ramanakumar A.V., Gilbert L., Reid S.L., Rodrigues I., Ferenczy A., Ratnam S., Coutlée F., Franco E.L. (2016). Human papillomavirus testing versus cytology in primary cervical cancer screening: End-of-study and extended follow-up results from the Canadian cervical cancer screening trial. Int. J. Cancer.

[87] Wentzensen N., Arbyn M. (2017). HPV-based cervical cancer screening- facts, fiction, and misperceptions. Prev. Med..

[88] Basu P., Meheus F., Chami Y., Hariprasad R., Zhao F., Sankaranarayanan R. (2017). Management algorithms for cervical cancer screening and precancer treatment for resource-limited settings. Int. J. Gynecol. Obstet..

[89] Jin X.W., Lipold L., Foucher J., Sikon A., Brainard J., Belinson J., Schramm S., Nottingham K., Hu B., Rothberg M.B. (2016). Cost-Effectiveness of Primary HPV Testing, Cytology and Co-testing as Cervical Cancer Screening for Women Above Age 30 Years. J. Gen. Intern. Med..

[90] Kothari A. (2017). The introduction of the HPV primary screening programme. Pract. Nurs..

[91] Tota J.E., Bentley J., Blake J., Coutlée F., Duggan M.A., Ferenczy A., Franco E.L., Fung-Kee-Fung M., Gotlieb W., Mayrand M.-H. (2017). Introduction of molecular HPV testing as the primary technology in cervical cancer screening: Acting on evidence to change the current paradigm. Prev. Med..

[92] Georgalis L., de Sanjosé S., Esnaola M., Bosch F.X., Diaz M. (2016). Present and future of cervical cancer prevention in Spain: A cost-effectiveness analysis. Eur. J. Cancer Prev..

[93] De Thurah L., Bonde J., Lam J.U.H., Rebolj M. (2018). Concordant testing results between various human papillomavirus assays in primary cervical cancer screening: Systematic review. Clin. Microbiol. Infect..

[94] Isidean S.D., Mayrand M.-H., Ramanakumar A.V., Rodrigues I., Ferenczy A., Ratnam S., Coutlée F., Franco E.L. (2017). Comparison of Triage Strategies for HPV-Positive Women: Canadian Cervical Cancer Screening Trial Results. Cancer Epidemiol. Biomarkers Prev..

[95] Schlichte M.J., Guidry J. (2015). Current Cervical Carcinoma Screening Guidelines. J. Clin. Med..

[96] Castañón A., Landy R., Cuzick J., Sasieni P. (2014). Cervical Screening at Age 50–64 Years and the Risk of Cervical Cancer at Age 65 Years and Older: Population-Based Case Control Study. PLoS Med..

[97] Gyllensten U., Lindell M., Gustafsson I., Wilander E. (2010). HPV test shows low sensitivity of Pap screen in older women. Lancet Oncol..

[98] Hermansson R.S., Olovsson M., Hoxell E., Lindström A.K. (2018). HPV prevalence and HPV-related dysplasia in elderly women. PLoS ONE.

[99] Naucler P., Ryd W., Törnberg S., Strand A., Wadell G., Elfgren K., Rådberg T., Strander B., Forslund O., Hansson B.-G. (2009). Efficacy of HPV DNA Testing With Cytology Triage and/or Repeat HPV DNA Testing in Primary Cervical Cancer Screening. JNCI J. Natl. Cancer Inst..

[100] Cuzick J., Wheeler C. (2016). Need for expanded HPV genotyping for cervical screening. Papillomavirus Res..

[101] Gustinucci D., Rossi P.G., Cesarini E., Broccolini M., Bulletti S., Carlani A., D’angelo V., D’amico M.R., Di Dato E., Galeazzi P. (2016). Use of Cytology, E6/E7 mRNA, and p16INK4a–Ki-67 to Define the Management of Human Papillomavirus (HPV)–Positive Women in Cervical Cancer Screening. Am. J. Clin. Pathol..

[102] Cuschieri K., Ronco G., Lorincz A., Smith L., Ogilvie G., Mirabello L., Carozzi F., Cubie H., Wentzensen N., Snijders P. (2018). Eurogin roadmap 2017: Triage strategies for the management of HPV-positive women in cervical screening programs. Int. J. Cancer.

[103] Cuzick J., Szarewski A., Cubie H., Hulman G., Kitchener H., Luesley D., McGoogan E., Menon U., Terry G., Edwards R. (2003). Management of women who test positive for high-risk types of human papillomavirus: The HART study. Lancet.

[104] Dijkstra M.G., van Niekerk D., Rijkaart D.C., van Kemenade F.J., Heideman D.A.M., Snijders P.J.F., Meijer C.J.L.M., Berkhof J. (2014). Primary hrHPV DNA Testing in Cervical Cancer Screening: How to Manage Screen-Positive Women? A POBASCAM Trial Substudy. Cancer Epidemiol. Biomarkers Prev..

[105] Ronco G., Dillner J., Elfström K.M., Tunesi S., Snijders P.J.F., Arbyn M., Kitchener H., Segnan N., Gilham C., Giorgi-Rossi P. (2014). Efficacy of HPV-based screening for prevention of invasive cervical cancer: Follow-up of four European randomised controlled trials. Lancet.

[106] Ronco G., Giorgi Rossi P. (2018). Role of HPV DNA testing in modern gynaecological practice. Best Pract. Res. Clin. Obstet. Gynaecol..

[107] Polman N.J., Veldhuijzen N.J., Heideman D.A.M., Snijders P.J.F., Meijer C.J.L.M., Berkhof J. (2017). HPV-positive women with normal cytology remain at increased risk of CIN3 after a negative repeat HPV test. Br. J. Cancer.

[108] Mariani L., Sandri M.T., Preti M., Origoni M., Costa S., Cristoforoni P., Bottari F., Sideri M. (2016). HPV-Testing in Follow-up of Patients Treated for CIN2+ Lesions. J. Cancer.

[109] Onuki M., Matsumoto K., Sakurai M., Ochi H., Minaguchi T., Satoh T., Yoshikawa H. (2016). Posttreatment human papillomavirus testing for residual or recurrent high-grade cervical intraepithelial neoplasia: A pooled analysis. J. Gynecol. Oncol..

[110] Cuschieri K., Bhatia R., Cruickshank M., Hillemanns P., Arbyn M. (2016). HPV testing in the context of post-treatment follow up (test of cure). J. Clin. Virol..

[111] Ribaldone R., Boldorini R., Capuano A., Arrigoni S., Di Oto A., Surico N. (2010). Role of HPV testing in the follow-up of women treated for cervical dysplasia. Arch. Gynecol. Obstet..

[112] Jordan J., Martin-Hirsch P., Arbyn M., Schenck U., Baldauf J.J., Silva D.D., Anttila A., Nieminen P., Prendiville W. (2009). European guidelines for clinical management of abnormal cervical cytology, Part 2. Cytopathology.

[113] Costa S., Venturoli S., Origoni M., Preti M., Mariani L., Cristoforoni P., Sandri M.T. (2015). Performance of HPV DNA testing in the follow-up after treatment of high-grade cervical lesions, adenocarcinoma in situ (AIS) and microinvasive carcinoma. Ecancermedicalscience.

[114] Asciutto K.C., Henic E., Darlin L., Forslund O., Borgfeldt C. (2016). Follow up with HPV test and cytology as test of cure, 6 months after conization, is reliable. Acta Obstet. Gynecol. Scand..

[115] Basu P., Ponti A., Anttila A., Ronco G., Senore C., Vale D.B., Segnan N., Tomatis M., Soerjomataram I., Primic Žakelj M. (2018). Status of implementation and organization of cancer screening in The European Union Member States—Summary results from the second European screening report. Int. J. Cancer.

[116] Stefan L., Terning H.B., Tor H., Suzanne C., Kristina S., Mari N. (2015). Cervical cancer prevented by screening: Long-term incidence trends by morphology in Norway. Int. J. Cancer.

[117] Paul K.T. (2016). “Saving lives”: Adapting and adopting Human Papilloma Virus (HPV) vaccination in Austria. Soc. Sci. Med..

[118] Anttila A., Ronco G. (2009). Description of the national situation of cervical cancer screening in the member states of the European Union. Eur. J. Cancer.

[119] Breitenecker G., Dinges H.P., Regitnig P., Wiener H., Vutuc C. (2004). Cytopathology in Austria. Cytopathology.

[120] Rásky É., Regitnig P., Schenouda M., Burkert N., Freidl W. (2013). Quality of screening with conventional Pap smear in Austria—A longitudinal evaluation. BMC Public Health.

[121] Schejter E., Bornstein J., Siegler E. (2013). Cervical Cancer Screening, Human Papillomavirus Vaccination Practices and Current Infrastructure in Israel. Vaccine.

[122] Bassal R., Schejter E., Bachar R., Shapira H., Kaufman Z., Cohen D., Keinan-Boker L. (2015). Recent trends of cervical cancer and Cervical Intraepithelial Neoplasia 3 (CIN3) in Israel. Arch. Gynecol. Obstet..

[123] Wymann M.N., Zographos A.S., Altpeter E., Spicher V.M., Low N., Mäusezahl-Feuz M. (2018). Human papillomavirus vaccine uptake in adolescence and adherence to cervical cancer screening in Switzerland: A national cross-sectional survey. Int. J. Public Health.

[124] Boiron L., Joura E., Largeron N., Prager B., Uhart M. (2016). Estimating the cost-effectiveness profile of a universal vaccination programme with a nine-valent HPV vaccine in Austria. BMC Infect. Dis..

[125] Altobelli E., Lattanzi A. (2015). Cervical Carcinoma in the European Union: An Update on Disease Burden, Screening Program State of Activation, and Coverage as of March 2014. Int. J. Gynecol. Cancer.

[126] Ponti A., Anttila A., Ronco G., Senore C., Basu P., Segnan N. (2017). Cancer Screening in the European Union (2017): Report on the Implementation of the Council Recommendation on Cancer Screening.

[127] Borena W., Grünberger M., Widschwendter A., Kraxner K.H., Marth E., Mayr P., Meier J., Ruth N., Guerrero A.T., Marth C. (2016). Pre-vaccine era cervical human papillomavirus infection among screening population of women in west Austria. BMC Public Health.

[128] Poncet L., Rigal L., Panjo H., Gautier A., Chauvin P., Menvielle G., Cadot E., Ringa V. (2016). Disengagement of general practitioners in cervical cancer screening. Eur. J. Cancer Prev..

[129] Paul K.T., Wallenburg I., Bal R. (2018). Putting public health infrastructures to the test: Introducing HPV vaccination in Austria and the Netherlands. Sociol. Health Illn..

[130] Lill C., Bachtiary B., Selzer E., Mittlboeck M., Thurnher D. (2017). A 5-year update of patients with HPV positive versus negative oropharyngeal cancer after radiochemotherapy in Austria. Wiener klinische Wochenschrift.

[131] World Health Organization Immunization, Vaccines and Biologicals. http://www.who.int/immunization/monitoring_surveillance/data/en/.

[132] Vandeweyer K., Tjalma W. (2017). PMD37—Cost-Effectiveness Analysis Of Primary HPV Screening With Dual-Stain Cytology Triage In The Cervical Cancer Screening Program Of Belgium. Value Health.

[133] Tjalma W.A., Kim E., Vandeweyer K. (2017). The impact on women’s health and the cervical cancer screening budget of primary HPV screening with dual-stain cytology triage in Belgium. Eur. J. Obstet. Gynecol. Reprod. Biol..

[134] Tjalma W.A.A., Trinh X.B., Rosenlund M., Makar A.P., Kridelka F., Rosillon D., Van Dam P.A., Collas De Souza S., Holl K., Simon P. (2015). A cross-sectional, multicentre, epidemiological study on human papillomavirus (HPV) type distribution in adult women diagnosed with invasive cervical cancer in Belgium. Facts Views Vis. ObGyn.

[135] Van Kerrebroeck H., Makar A. (2016). Cervical cancer screening in Belgium and overscreening of adolescents. Eur. J. Cancer Prev..

[136] Coorevits L., Traen A., Bingé L., Van Dorpe J., Praet M., Boelens J., Padalko E. (2018). Are vaginal swabs comparable to cervical smears for human papillomavirus DNA testing?. J. Gynecol. Oncol..

[137] Flemish Minister for Welfare, Public Health and Family Centrum Voor Kankeropsporing-CvKO. https://baarmoederhalskanker.bevolkingsonderzoek.be/nl/wij-doen-het-en-wat-doe-jij.

[138] Arbyn M., Broeck D.V., Benoy I., Bogers J., Depuydt C., Praet M., De Sutter P., Hoorens A., Hauben E., Poppe W. (2016). Surveillance of effects of HPV vaccination in Belgium. Cancer Epidemiol..

[139] Arbyn M., Haelens A., Desomer A., Verdoodt F., Thiry N., Francart J., Hanquet G., Robays J. (2015). Cervical Cancer Screening Program and Human Papillomavirus (HPV) Testing, Part II: Update on HPV Primary Screening.

[140] Lefevere E., Theeten H., Hens N., De Smet F., Top G., Van Damme P. (2015). From non school-based, co-payment to school-based, free Human Papillomavirus vaccination in Flanders (Belgium): A retrospective cohort study describing vaccination coverage, age-specific coverage and socio-economic inequalities. Vaccine.

[141] Lefevere E., Hens N., Theeten H., Van den Bosch K., Beutels P., De Smet F., Van Damme P. (2011). Like mother, like daughter? Mother’s history of cervical cancer screening and daughter’s Human Papillomavirus vaccine uptake in Flanders (Belgium). Vaccine.

[142] Wentzensen N., Arbyn M., Berkhof J., Bower M., Canfell K., Einstein M., Farley C., Monsonego J., Franceschi S. (2017). Eurogin 2016 Roadmap: How HPV knowledge is changing screening practice. Int. J. Cancer.

[143] Tjalma W.A.A. (2017). Diagnostic performance of dual-staining cytology for cervical cancer screening: A systematic literature review. Eur. J. Obstet. Gynecol. Reprod. Biol..

[144] Kesic V., Poljak M., Rogovskaya S. (2012). Cervical cancer burden and prevention activities in Europe. Cancer Epidemiol. Prev. Biomarkers.

[145] Todorova I., Alexandrova-Karamanova A., Panayotova Y., Dimitrova E., Kotzeva T. (2014). Managing uncertainty: Healthcare professionals’ meanings regarding the HPV vaccine. Int. J. Behav. Med..

[146] Samson K.K., Haynatzki G., Soliman A.S., Valerianova Z. (2016). Temporal changes in the cervical cancer burden in Bulgaria: Implications for eastern european countries going through transition. Cancer Epidemiol..

[147] Maver P.J., Seme K., Korac T., Dimitrov G., Dobrossy L., Engele L., Iljazovic E., Kesic V., Kostova P., Lausevic D. (2013). Cervical cancer screening practices in central and eastern Europe in 2012. Acta Dermatovenerol. Alp. Pannonica Adriat..

[148] Vaccarella S., Franceschi S., Zaridze D., Poljak M., Veerus P., Plummer M., Bray F. (2016). Preventable fractions of cervical cancer via effective screening in six Baltic, central, and eastern European countries 2017–40: A population-based study. Lancet Oncol..

[149] Sabol I., Milutin Gašperov N., Matovina M., Božinović K., Grubišić G., Fistonić I., Belci D., Alemany L., Džebro S., Dominis M. (2017). Cervical HPV type-specific pre-vaccination prevalence and age distribution in Croatia. PLoS ONE.

[150] Štemberger-Papić S., Vrdoljak-Mozetič D., Verša Ostojić D., Rubeša-Mihaljević R., Dinter M. (2016). Cervical cytology (Pap test)–terminology and importance in screening for cervical cancer. Med. Flum..

[151] Krashias G., Koptides D., Christodoulou C. (2017). HPV prevalence and type distribution in Cypriot women with cervical cytological abnormalities. BMC Infect. Dis..

[152] Papapetrou I., Charalambous G., Sissouras A., Jelastopulu E. (2016). Cervical Cancer Screening in the Municipality of Nicosia, Cyprus-Coverage and Association with Socioeconomic Determinants. Austin J Public Health Epidemiol..

[153] Farazi P.A. (2014). Cancer trends and risk factors in Cyprus. Ecancermedicalscience.

[154] Charalambous H. (2016). Cancer Care in an Economically Torn Country: Cyprus. Cancer Care in Countries and Societies in Transition.

[155] Májek O., Dušková J., Dvorák V., Beková A., Klimeš D., Blaha M., Anttila A., Dušek L. (2017). Performance indicators in a newly established organized cervical screening programme: Registry-based analysis in the Czech Republic. Eur. J. Cancer Prev..

[156] Májek O., Dvořák V., Dušek L., Mužík J., Šnajdrová L., Gregor J. Cervix. cz–Proč Pozvala Vaše Zdravotní Pojišťovna Právě Vás?. http://www.cervix.cz/index.php?pg=cervikalni-screening--adresne-zvani-obcanu-do-programu-screeningu-zhoubnych-nadoru--proc-prave-vy#top.

[157] Dušková J., Beková A., Dvořák V., Májek O., Dušek L. (2014). Results of the Czech National Cervical Cancer screening programme. Klinicka Onkologie: Casopis Ceske a Slovenske Onkologicke Spolecnosti.

[158] Hamsikova E., Smahelova J., Ludvikova V., Salakova M., Rychla J., Skrenkova J., Rob L., Tachezy R. (2017). The prevalence of HPV infections in HPV-vaccinated women from the general population. APMIS.

[159] Brotherton J.M.L., Zuber P.L.F., Bloem P.J.N. (2016). Primary Prevention of HPV through Vaccination: Update on the Current Global Status. Curr. Obstet. Gynecol. Rep..

[160] Elfström K.M., Dillner J., Arnheim-Dahlström L. (2015). Organization and quality of HPV vaccination programs in Europe. Vaccine.

[161] Rygaard C. (2016). The Danish Quality Database for Cervical Cancer Screening. Clin. Epidemiol..

[162] Lynge E., Andersen B., Christensen J., Ejersbo D., Jochumsen K., Johansen T., Kristensen J.K., Larsen L.G., Mehnert F., Mikkelsen E. (2018). Cervical screening in Denmark—A success followed by stagnation. Acta Oncol..

[163] Kristiansen B.K., Andersen B., Bro F., Svanholm H., Vedsted P. (2017). Impact of GP reminders on follow-up of abnormal cervical cytology: A before–after study in Danish general practice. Br. J. Gen. Pract..

[164] Azerkan F., Widmark C., Sparén P., Weiderpass E., Tillgren P., Faxelid E. (2015). When Life Got in the Way: How Danish and Norwegian Immigrant Women in Sweden Reason about Cervical Screening and Why They Postpone Attendance. PLoS ONE.

[165] Baandrup L., Blomberg M., Dehlendorff C., Sand C., Andersen K.K., Kjaer S.K. (2013). Significant decrease in the incidence of genital warts in young Danish women after implementation of a national human papillomavirus vaccination program. Sex. Transm. Dis..

[166] Skorstengaard M., Thamsborg L.H., Lynge E. (2017). Burden of HPV-caused cancers in Denmark and the potential effect of HPV-vaccination. Vaccine.

[167] Olsen J., Jørgensen T.R. (2015). Revisiting the cost-effectiveness of universal HPV-vaccination in Denmark accounting for all potentially vaccine preventable HPV-related diseases in males and females. Cost Eff. Resour. Alloc..

[168] Hariri S., Markowitz L.E., Dunne E.F., Unger E.R. (2013). Population impact of HPV vaccines: Summary of early evidence. J. Adolesc. Health.

[169] Kempers J., Narvestad J., Kofod M., Mikkelsen R. (2016). Budget Impact Analysis of National Cervical Cancer Screening Program in Denmark: Cytology with HPV triage vs. HPV Primary Screening with Reflex Cytology Triage & Cintec Plus Cytology. Value Health.

[170] Rebolj M., Njor S., Lynge E., Preisler S., Ejegod D., Rygaard C., Bonde J. (2017). Referral population studies underestimate differences between human papillomavirus assays in primary cervical screening. Cytopathology.

[171] Von Euler-Chelpin M., Lynge E., Rebolj M. (2011). Register-based studies of cancer screening effects. Scand. J. Public Health.

[172] Kivistik A., Lang K., Baili P., Anttila A., Veerus P. (2011). Women’s knowledge about cervical cancer risk factors, screening, and reasons for non-participation in cervical cancer screening programme in Estonia. BMC Womens Health.

[173] Uusküla A., Müürsepp A., Kawai K., Raag M., Jürisson M., Pillsbury M. (2013). The epidemiological and economic impact of a quadrivalent human papillomavirus (hpv) vaccine in Estonia. BMC Infect. Dis..

[174] Võrno T., Lutsar K., Uusküla A., Padrik L., Raud T., Reile R., Nahkur O., Kiivet R.-A. (2017). Cost-effectiveness of HPV vaccination in the context of high cervical cancer incidence and low screening coverage. Vaccine.

[175] Malila N., Leinonen M., Kotaniemi-Talonen L., Laurila P., Tarkkanen J., Hakama M. (2013). The HPV test has similar sensitivity but more overdiagnosis than the Pap test—A randomised health services study on cervical cancer screening in Finland. Int. J. Cancer.

[176] Lönnberg S., Anttila A., Luostarinen T., Nieminen P. (2012). Age-specific effectiveness of the Finnish cervical cancer screening programme. Cancer Epidemiol. Prev. Biomarkers.

[177] Makkonen P., Heinävaara S., Sarkeala T., Anttila A. (2017). Impact of organized and opportunistic Pap testing on the risk of cervical cancer in young women—A case-control study from Finland. Gynecol. Oncol..

[178] Veijalainen O., Tuomisaari S., Luukkaala T., Mäenpää J. (2015). High risk HPV testing in the triage of repeat ASC-US and LSIL. Acta Obstetr. Gynecol. Scand..

[179] Idehen E.E., Korhonen T., Castaneda A., Juntunen T., Kangasniemi M., Pietilä A.-M., Koponen P. (2017). Factors associated with cervical cancer screening participation among immigrants of Russian, Somali and Kurdish origin: A population-based study in Finland. BMC Womens Health.

[180] Van der Aa M.A., Pukkala E., Coebergh J.W.W., Anttila A., Siesling S. (2008). Mass screening programmes and trends in cervical cancer in Finland and the Netherlands. Int. J. Cancer.

[181] Anttila A., Kotaniemi-Talonen L., Leinonen M., Hakama M., Laurila P., Tarkkanen J., Malila N., Nieminen P. (2010). Rate of cervical cancer, severe intraepithelial neoplasia, and adenocarcinoma in situ in primary HPV DNA screening with cytology triage: Randomised study within organised screening programme. BMJ.

[182] Skufca J., Ollgren J., Ruokokoski E., Lyytikäinen O., Nohynek H. (2017). Incidence rates of Guillain Barré (GBS), chronic fatigue/systemic exertion intolerance disease (CFS/SEID) and postural orthostatic tachycardia syndrome (POTS) prior to introduction of human papilloma virus (HPV) vaccination among adolescent girls in Finland, 2002–2012. Papillomavirus Res..

[183] Virtanen A., Anttila A., Luostarinen T., Nieminen P. (2011). Self-sampling versus reminder letter: Effects on cervical cancer screening attendance and coverage in Finland. Int. J. Cancer.

[184] Karjalainen L., Anttila A., Nieminen P., Luostarinen T., Virtanen A. (2016). Self-sampling in cervical cancer screening: Comparison of a brush-based and a lavage-based cervicovaginal self-sampling device. BMC Cancer.

[185] Hamers F.F., Duport N., Beltzer N. (2018). Population-based organized cervical cancer screening pilot program in France. Eur. J. Cancer Prev..

[186] Barré S., Massetti M., Leleu H., De Bels F. (2017). Organised screening for cervical cancer in France: A cost-effectiveness assessment. BMJ Open.

[187] Uhart M., Adam M., Dahlab A., Bresse X. (2017). Loss of chance associated with sub-optimal HPV vaccination coverage rate in France. Papillomavirus Res..

[188] Schaffer P., Sancho-Garnier H., Fender M., Dellenbach P., Carbillet J., Monnet E., Gauthier G., Garnier A. (2000). Cervical cancer screening in France. Eur. J. Cancer.

[189] Miranda S., Chaignot C., Collin C., Dray-Spira R., Weill A., Zureik M. (2017). Human papillomavirus vaccination and risk of autoimmune diseases: A large cohort study of over 2 million young girls in France. Vaccine.

[190] Fagot J.-P., Boutrelle A., Ricordeau P., Weill A., Allemand H. (2011). HPV vaccination in France: Uptake, costs and issues for the National Health Insurance. Vaccine.

[191] Shield K.D., Marant Micallef C., de Martel C., Heard I., Megraud F., Plummer M., Vignat J., Bray F., Soerjomataram I. (2018). New cancer cases in France in 2015 attributable to infectious agents: A systematic review and meta-analysis. Eur. J. Epidemiol..

[192] Guenat D., Launay S., Riethmuller D., Mougin C., Prétet J.-L. (2016). Validation of Novaprep^®^ HQ+ liquid-based cytology medium for high-risk human papillomavirus detection by hc2. Infect. Agents Cancer.

[193] Radde K., Gottschalk A., Bussas U., Schülein S., Schriefer D., Seifert U., Neumann A., Kaiser M., Blettner M., Klug S.J. (2016). Invitation to cervical cancer screening does increase participation in Germany: Results from the MARZY study. Int. J. Cancer.

[194] Garbe Y., Klug S.J. (2017). Aktueller Stand der HPV-Impfung in Deutschland. Der Onkologe.

[195] Geyer S., Jaunzeme J., Hillemanns P. (2015). Cervical cancer screening in Germany: Group-specific participation rates in the state of Niedersachsen (Lower Saxony). A study with health insurance data. Arch. Gynecol. Obstet..

[196] Damm O., Horn J., Mikolajczyk R.T., Kretzschmar M.E.E., Kaufmann A.M., Deleré Y., Ultsch B., Wichmann O., Krämer A., Greiner W. (2017). Cost-effectiveness of human papillomavirus vaccination in Germany. Cost Eff. Resour. Alloc..

[197] Hillemanns P. (2016). The paradigm shift in cervical cancer screening in Germany. Arch. Gynecol. Obstet..

[198] Schneider V. (2012). Gynäkologische Krebsvorsorge in Deutschland. Der Pathologe.

[199] Schmidt D., Neumann H.H. (2015). Wer macht die gynäkologische Zytologie und wie?. Der Pathologe.

[200] Horn J., Damm O., Kretzschmar M.E.E., Deleré Y., Wichmann O., Kaufmann A.M., Garbe E., Krämer A., Greiner W., Mikolajczyk R.T. (2013). Estimating the long-term effects of HPV vaccination in Germany. Vaccine.

[201] Petry K.U., Barth C., Wasem J., Neumann A. (2017). A model to evaluate the costs and clinical effectiveness of human papilloma virus screening compared with annual papanicolaou cytology in Germany. Eur. J. Obstet. Gynecol. Reprod. Biol..

[202] Farazi P.A., Hadji P., Roupa Z. (2017). Awareness of human papilloma virus and cervical cancer prevention among Greek female healthcare workers. Eur. J. Cancer Prev..

[203] Bacopoulou F., Karakitsos P., Kottaridi C., Stefanaki C., Deligeoroglou E., Theodoridou K., Chrousos G.P., Michos A. (2016). Genital HPV in Children and Adolescents: Does Sexual Activity Make a Difference?. J. Pediatr. Adolesc. Gynecol..

[204] Chatzistamatiou K., Chatzaki E., Constantinidis T., Nena E., Tsertanidou A., Agorastos T. (2017). Self-collected cervicovaginal sampling for site-of-care primary HPV-based cervical cancer screening: A pilot study in a rural underserved Greek population. J. Obstet. Gynaecol..

[205] Vaidakis D., Moustaki I., Zervas I., Barbouni A., Merakou K., Chrysi M.S., Creatsa G., Panoskaltsis T. (2017). Knowledge of Greek adolescents on human papilloma virus (HPV) and vaccination: A national epidemiologic study. Medicine.

[206] Jelastopulu E., Karnaki P., Bartsokas C., Plotas P., Sissouras A. (2013). Screening for Cervical Cancer-Uptake and Associated Factors in a Representative Sample in the City of Patras, West-Greece. Univers. J. Public Health.

[207] Karamanidou C., Dimopoulos K. (2016). Greek health professionals’ perceptions of the HPV vaccine, state policy recommendations and their own role with regards to communication of relevant health information. BMC Public Health.

[208] Gyulai A., Nagy A., Pataki V., Tonté D., Ádány R., Vokó Z. (2015). Survey of participation in organised cervical cancer-screening programme in hungary. Cent. Eur. J. Public Health.

[209] Balla B., Terebessy A., Tóth E., Balázs P. (2016). Young Hungarian Students’ Knowledge about HPV and Their Attitude Toward HPV Vaccination. Vaccines.

[210] Pakai A., Brantmüller É., Réka V., Karácsony I., Balázs P. (2016). Reasons for Non-appearance on Organized Cervical Screening in Hungary. Pract. Theory Syst. Educ..

[211] Döbrőssy L., Oroszi B., Kovács A., Budai A. (2016). Comprehensive Programme to Prevent Cervical Cancer-the Case of Hungary. Int. J. Virol. AIDS.

[212] Marek E., Berenyi K., Dergez T., Kiss I., D’Cruz G. (2016). Influence of risk-taking health behaviours of adolescents on cervical cancer prevention: A Hungarian survey. Eur. J. Cancer Care.

[213] Szentirmay Z., Veleczki Z., Kásler M. (2017). Human papillomavirus associated cervix uteri morbidity in Hungary: Epidemiology and correlation with the HPV types and the simultaneous cytological diagnosis. Orv. Hetil..

[214] Vokó Z., Nagyjánosi L., Margitai B., Kövi R., Tóth Z., László D., Kaló Z. (2012). Modeling Cost-Effectiveness of Cervical Cancer Screening in Hungary. Value Health.

[215] Tsonev A., Ivanov S., Kovachev E. (2013). Liquid-based cytology and its practicability in Bulgaria. Scr. Sci. Med..

[216] McCarthy C.M., Ramphul M., Madden M., Hickey K. (2016). The use and success of cold coagulation for the treatment of high grade squamous cervical intra-epithelial neoplasia: A retrospective review. Eur. J. Obstet. Gynecol. Reprod. Biol..

[217] Flannelly G.M., Mooney M.T., Greehy G.M., Keogh E.B., McNally S.A., Fitzpatrick P.E. (2018). Establishment of a national cervical screening programme in Ireland, CervicalCheck: The first 6 years. Eur. J. Cancer Prev..

[218] Teljeur C., Tyrrell E., Kelly A., O’Dowd T., Thomas S. (2014). Getting a handle on the general practice workforce in Ireland. Ir. J. Med. Sci..

[219] O’Connor M., Costello L., Murphy J., Prendiville W., Martin C.M., O’Leary J.J., Sharp L., Irish Screening Research Consortium (2014). ‘I don’t care whether it’s HPV or ABC, I just want to know if I have cancer.’ Factors influencing women’s emotional responses to undergoing human papillomavirus testing in routine management in cervical screening: A qualitative study. BJOG Int. J. Obstet. Gynaecol..

[220] Giorgi Rossi P., Carozzi F., Federici A., Ronco G., Zappa M., Franceschi S. (2017). Cervical cancer screening in women vaccinated against human papillomavirus infection: Recommendations from a consensus conference. Prev. Med..

[221] Di Stefano F., Giorgi Rossi P., Carozzi F., Ronco G., Cacciani L., Vecchi S., Naldoni C., Segnan N., Gruppo di Lavoro MIDDIR—HPV Test In Primary Screening (2017). [Implementation of DNA-HPV primary screening in Italian cervical cancer screening programmes. Results of the MIDDIR Project]. Epidemiol. Prev..

[222] Ronco G., Zappa M., Franceschi S., Tunesi S., Caprioglio A., Confortini M., Del Mistro A., Carozzi F., Segnan N., Zorzi M. (2016). Impact of variations in triage cytology interpretation on human papillomavirus–based cervical screening and implications for screening algorithms. Eur. J. Cancer.

[223] Bucchi L., Cristiani P., Costa S., Schincaglia P., Garutti P., Sassoli de Bianchi P., Naldoni C., Olea O., Sideri M. (2013). Rationale and development of an on-line quality assurance programme for colposcopy in a population-based cervical screening setting in Italy. BMC Health Serv. Res..

[224] Pasquale L., Rossi P.G., Carozzi F., Pedretti C., Ruggeri C., Scalvinoni V., Cottini M.C., Tosini A., Morana C., Chiaramonte M. (2015). Cervical cancer screening with HPV testing in the Valcamonica (Italy) screening programme. J. Med. Screen..

[225] Maggino T., Sciarrone R., Murer B., Dei Rossi M.R., Fedato C., Maran M., Lorio M., Soldà M., Zago F., Rossi P.G. (2016). Screening women for cervical cancer carcinoma with a HPV mRNA test: First results from the Venice pilot program. Br. J. Cancer.

[226] Carozzi F.M., Iossa A., Scalisi A., Sideri M., Andersson K.L., Confortini M., Del Mistro A., Maina G., Ronco G., Raggi P. (2015). hr-HPV testing in the management of women with ASC-US+ and in the follow-up of women with cytological abnormalities and negative colposcopy. Recommendations of the Italian group for cervical cancer screening (GISCi). Epidemiol. Prev..

[227] Carozzi F., Visioli C.B., Confortini M., Iossa A., Mantellini P., Burroni E., Zappa M. (2013). hr-HPV testing in the follow-up of women with cytological abnormalities and negative colposcopy. Br. J. Cancer.

[228] Vīberga I., Poljak M. (2013). Cervical cancer screening in Latvia: A brief history and recent improvements (2009–2011). Acta Dermatovenerol. Alp. Pannonica Adriat..

[229] Kornete A., Pumpure E., Macuks R. (2016). Analysis of invasive cervical cancer cases in Latvia. Int. J. Reprod. Contracept. Obstet. Gynecol..

[230] Viberga I., Engele L., Kojalo U., Santare D. (2014). Professionals’ role in implementing a cervical cancer screening program. Acta Dermatovenerol. Alp. Pannonica Adriat..

[231] Patel H., Pčolkina K., Strazdina K., Viberga I., Sherman S.M., Tincello D.G., Redman C.W., Rezeberga D., Moss E.L. (2017). Awareness of HPV infection and attitudes toward HPV vaccination among Latvian adolescents. Int. J. Gynecol. Obstet..

[232] Kurtinaitienė R., Rimienė J., Labanauskaitė I., Lipunova N., Smailytė G. (2016). Increasing attendance in a cervical cancer screening programme by personal invitation: Experience of a Lithuanian primary health care centre. Acta Med. Litu..

[233] Kurtinaitienė R., Drąsutienė G., Labanauskaitė I., Akelytė A., Drąsutytė L. (2008). Vilniaus miesto moterų žinios apie gimdos kaklelio vėžio rizikos veiksnius ir patikros programą. MTP.

[234] Latsuzbaia A., Hebette G., Fischer M., Arbyn M., Weyers S., Vielh P., Schmitt F., Mossong J. (2017). Introduction of liquid-based cytology and human papillomavirus testing in cervical cancer screening in Luxembourg. Diagn. Cytopathol..

[235] Sankaranarayanan R., Qiao Y.-L., Keita N. (2015). The Next Steps in Cervical Screening. Women’s Health.

[236] Scheiden R., Knolle U., Wagener C., Wehenkel A.M., Capesius C. (2000). Cervical cancer screening in Luxembourg. Eur. J. Cancer.

[237] Scheiden R., Wagener C., Knolle U., Wehenkel A., Dippel W., Capesius C. (2003). Cervical screening in Luxembourg: 1990–1999. Cytopathology.

[238] Anttila A., von Karsa L., Aasmaa A., Fender M., Patnick J., Rebolj M., Nicula F., Vass L., Valerianova Z., Voti L. (2009). Cervical cancer screening policies and coverage in Europe. Eur. J. Cancer.

[239] Latsuzbaia A., Tapp J., Nguyen T., Fischer M., Arbyn M., Weyers S., Mossong J. (2016). Analytical performance evaluation of Anyplex II HPV28 and Euroarray HPV for genotyping of cervical samples. Diagn. Microbiol. Infect. Dis..

[240] Government of Malta Cervix Screening Programme in Malta. https://deputyprimeminister.gov.mt/en/phc/nbs/Pages/Cervix-Screening-Programme.aspx.

[241] Huijsmans C.J.J., Geurts-Giele W.R.R., Leeijen C., Hazenberg H.L.C.M., van Beek J., de Wild C., van der Linden J.C., van den Brule A.J.C. (2016). HPV Prevalence in the Dutch cervical cancer screening population (DuSC study): HPV testing using automated HC2, cobas and Aptima workflows. BMC Cancer.

[242] Van Ballegooijen M., Hermens R. (2000). Cervical cancer screening in The Netherlands. Eur. J. Cancer.

[243] Rozemeijer K., de Kok I.M.C.M., Naber S.K., van Kemenade F.J., Penning C., van Rosmalen J., van Ballegooijen M. (2015). Offering Self-Sampling to Non-Attendees of Organized Primary HPV Screening: When Do Harms Outweigh the Benefits?. Cancer Epidemiol. Biomarkers Prev..

[244] Ketelaars P.J.W., Bosgraaf R.P., Siebers A.G., Massuger L.F.A.G., van der Linden J.C., Wauters C.A.P., Rahamat-Langendoen J.C., van den Brule A.J.C., IntHout J., Melchers W.J.G. (2017). High-risk human papillomavirus detection in self-sampling compared to physician-taken smear in a responder population of the Dutch cervical screening: Results of the VERA study. Prev. Med..

[245] Qendri V., Bogaards J.A., Berkhof J. (2017). Health and Economic Impact of a Tender-Based, Sex-Neutral Human Papillomavirus 16/18 Vaccination Program in the Netherlands. J. Infect. Dis..

[246] Naber S.K., Matthijsse S.M., Rozemeijer K., Penning C., de Kok I.M.C.M., van Ballegooijen M. (2016). Cervical Cancer Screening in Partly HPV Vaccinated Cohorts—A Cost-Effectiveness Analysis. PLoS ONE.

[247] Nowakowski A., Cybulski M., Śliwczyński A., Chil A., Teter Z., Seroczyński P., Arbyn M., Anttila A. (2015). The implementation of an organised cervical screening programme in Poland: An analysis of the adherence to European guidelines. BMC Cancer.

[248] Nowakowski A., Wojciechowska U., Wieszczy P., Cybulski M., Kamiński M.F., Didkowska J. (2017). Trends in cervical cancer incidence and mortality in Poland: Is there an impact of the introduction of the organised screening?. Eur. J. Epidemiol..

[249] Kalinowski P., Grządziel A. (2017). HPV Vaccinations in Lublin Region, Poland. Postepy Hig. Med. Doswiadczalnej (Online).

[250] Nowakowski A., de Souza S.C., Jach R., Rosillon D., Książek A., Holl K. (2015). HPV-Type Distribution and Reproducibility of Histological Diagnosis in Cervical Neoplasia in Poland. Pathol. Oncol. Res..

[251] Costa A.R., Silva S., Moura-Ferreira P., Villaverde-Cabral M., Santos O., do Carmo I., Barros H., Lunet N. (2017). Cancer screening in Portugal: Sex differences in prevalence, awareness of organized programmes and perception of benefits and adverse effects. Health Expect..

[252] Real O., Silva D., Leitão M.A., Oliveira H.M., Rocha Alves J.G. (2000). Cervical cancer screening in the central region of Portugal. Eur. J. Cancer.

[253] Firmino-Machado J., Mendes R., Moreira A., Lunet N. (2017). Stepwise strategy to improve Cervical Cancer Screening Adherence (SCAN-CC): Automated text messages, phone calls and face-to-face interviews: Protocol of a population-based randomised controlled trial. BMJ Open.

[254] Pista A., de Oliveira C.F., Lopes C., Cunha M.J., CLEOPATRE Portugal Study Group (2017). Potential impact of nonavalent HPV vaccine in the prevention of high-grade cervical lesions and cervical cancer in Portugal. Int. J. Gynecol. Obstet..

[255] Grigore M., Popovici R., Pristavu A., Grigore A.M., Matei M., Gafitanu D. (2017). Perception and use of Pap smear screening among rural and urban women in Romania. Eur. J. Public Health.

[256] Penţa M.A., Băban A. (2014). Mass media coverage of HPV vaccination in Romania: A content analysis. Health Educ. Res..

[257] Vorsters A., Arbyn M., Baay M., Bosch X., de Sanjosé S., Hanley S., Karafillakis E., Lopalco P.L., Pollock K.G., Yarwood J. (2017). Overcoming barriers in HPV vaccination and screening programs. Papillomavirus Res..

[258] Grigore M., Teleman S.I., Pristavu A., Matei M. (2018). Awareness and Knowledge About HPV and HPV Vaccine Among Romanian Women. J. Cancer Educ..

[259] Craciun C., Baban A. (2012). “Who will take the blame?”: Understanding the reasons why Romanian mothers decline HPV vaccination for their daughters. Vaccine.

[260] Andreassen T., Melnic A., Figueiredo R., Moen K., Şuteu O., Nicula F., Ursin G., Weiderpass E. (2018). Attendance to cervical cancer screening among Roma and non-Roma women living in North-Western region of Romania. Int. J. Public Health.

[261] Obročníková A., Majerníková Ľ. (2017). Knowledge, attitudes and practices of cervical cancer prevention. Pielegniarstwo XXI Wieku/Nurs. 21st Century.

[262] Jackowska M., von Wagner C., Wardle J., Juszczyk D., Luszczynska A., Waller J. (2012). Cervical screening among migrant women: A qualitative study of Polish, Slovak and Romanian women in London, UK. J. Fam. Plan. Reprod. Health Care.

[263] Bastos J., Peleteiro B., Gouveia J., Coleman M.P., Lunet N. (2010). The state of the art of cancer control in 30 European countries in 2008. Int. J. Cancer.

[264] Rajčáni J., Kajo K., Hassoun O.E., Adamkov M., Benčat M., Rajkumar R. (2016). The Diagnostic of Cervical Carcinoma: From Theory to Practice. Human Papillomavirus—Research in a Global Perspective.

[265] Jančar N., Mihevc Ponikvar B., Tomšič S. (2016). Cold-knife conisation and large loop excision of transformation zone significantly increase the risk for spontaneous preterm birth: A population-based cohort study. Eur. J. Obstet. Gynecol. Reprod. Biol..

[266] Zadnik V., Primic Zakelj M., Lokar K., Jarm K., Ivanus U., Zagar T. (2017). Cancer burden in slovenia with the time trends analysis. Radiol. Oncol..

[267] Učakar V., Jelen M.M., Faust H., Poljak M., Dillner J., Klavs I. (2013). Pre-vaccination seroprevalence of 15 human papillomavirus (HPV) types among women in the population-based Slovenian cervical screening program. Vaccine.

[268] Šubelj M., Učakar V., Kraigher A., Klavs I., Prevention (2016). Adverse events following school-based vaccination of girls with quadrivalent human papillomavirus vaccine in Slovenia, 2009 to 2013. Euro Surveill..

[269] Marzo-Castillejo M., Bellas-Beceiro B., Vela-Vallespín C., Nuin-Villanueva M., Bartolomé-Moreno C., Melús-Palazón E., Vilarrubí-Estrella M. (2016). Recomendaciones de prevención del cáncer. Actualización 2016. Aten. Primaria.

[270] Trapero-Bertran M., Acera Pérez A., de Sanjosé S., Manresa Domínguez J.M., Rodríguez Capriles D., Rodriguez Martinez A., Bonet Simó J.M., Sanchez Sanchez N., Hidalgo Valls P., Díaz Sanchis M. (2017). Cost-effectiveness of strategies to increase screening coverage for cervical cancer in Spain: The CRIVERVA study. BMC Public Health.

[271] Fernández Calvo M.T., Hernández Rubio A., Rosell Aguilar I. (2000). Cervical cancer screening in Spain. Eur. J. Cancer.

[272] Limia A., Pachón I. (2011). Coverage of human papillomavirus vaccination during the first year of its introduction in Spain. Euro Surveill..

[273] Ibáñez R., Moreno-Crespi J., Sardà M., Autonell J., Fibla M., Gutiérrez C., Lloveras B., Alejo M., Català I., Alameda F. (2012). Prediction of cervical intraepithelial neoplasia grade 2+ (CIN2+) using HPV DNA testing after a diagnosis of atypical squamous cell of undetermined significance (ASC-US) in Catalonia, Spain. BMC Infect. Dis..

[274] Yuan L., Hu Y., Zhou Z., Gong Y., Wang R., Li N. (2017). Quantitative methylation analysis to detect cervical (pre)-cancerous lesions in high-risk HPV-positive women. Int. J. Clin. Exp. Med..

[275] Pérez-Castro S., Lorenzo-Mahía Y., Iñarrea Fernández A., Lamas-González M.J., Sarán-Díez M.T., Rubio-Alarcón J., Reboredo-Reboredo M.C., Mosteiro-Lobato S., López-Miragaya I., Torres-Piñón J. (2014). Cervical intraepithelial neoplasia grade 2 or worse in Galicia, Spain: HPV 16 prevalence and vaccination impact. Enferm. Infecc. Microbiol. Clín..

[276] Castillo M., Astudillo A., Clavero O., Velasco J., Ibáñez R., de Sanjosé S. (2016). Poor Cervical Cancer Screening Attendance and False Negatives. A Call for Organized Screening. PLoS ONE.

[277] Cervantes-Amat M., López-Abente G., Aragonés N., Pollán M., Pastor-Barriuso R., Pérez-Gómez B. (2015). The end of the decline in cervical cancer mortality in Spain: Trends across the period 1981–2012. BMC Cancer.

[278] Ascunce N., Salas D., Zubizarreta R., Almazán R., Ibáñez J., Ederra M. (2010). Cancer screening in Spain. Ann. Oncol..

[279] Östensson E., Fröberg M., Leval A., Hellström A.-C., Bäcklund M., Zethraeus N., Andersson S. (2015). Cost of Preventing, Managing, and Treating Human Papillomavirus (HPV)-Related Diseases in Sweden before the Introduction of Quadrivalent HPV Vaccination. PLoS ONE.

[280] Baltzer N., Sundström K., Nygård J.F., Dillner J., Komorowski J. (2017). Risk stratification in cervical cancer screening by complete screening history: Applying bioinformatics to a general screening population. Int. J. Cancer.

[281] Andrae B., Kemetli L., Sparén P., Silfverdal L., Strander B., Ryd W., Dillner J., Törnberg S. (2008). Screening-Preventable Cervical Cancer Risks: Evidence From a Nationwide Audit in Sweden. JNCI J. Natl. Cancer Inst..

[282] Alfonzo E., Andersson Ellström A., Nemes S., Strander B. (2016). Effect of Fee on Cervical Cancer Screening Attendance—ScreenFee, a Swedish Population-Based Randomised Trial. PLoS ONE.

[283] Thomsen L.T., Nygård M., Stensen S., Terning Hansen B., Arnheim Dahlström L., Liaw K.-L., Munk C., Kjaer S.K. (2017). Awareness of human papillomavirus after introduction of HPV vaccination: A large population-based survey of Scandinavian women. Eur. J. Cancer Prev..

[284] Hortlund M., Sundström K., Lamin H., Hjerpe A., Dillner J. (2016). Laboratory audit as part of the quality assessment of a primary HPV-screening program. J. Clin. Virol..

[285] Lamin H., Eklund C., Elfström K.M., Carlsten-Thor A., Hortlund M., Elfgren K., Törnberg S., Dillner J. (2017). Randomised healthcare policy evaluation of organised primary human papillomavirus screening of women aged 56–60. BMJ Open.

[286] Pedersen K., Fogelberg S., Thamsborg L.H., Clements M., Nygård M., Kristiansen I.S., Lynge E., Sparén P., Kim J.J., Burger E.A. (2018). An overview of cervical cancer epidemiology and prevention in Scandinavia. Acta Obstet. Gynecol. Scand.

[287] Aref-Adib M., Freeman-Wang T. (2016). Cervical cancer prevention and screening: The role of human papillomavirus testing. Obstet. Gynaecol..

[288] Albrow R., Kitchener H., Gupta N., Desai M. (2012). Cervical screening in England: The past, present, and future. Cancer Cytopathol..

[289] Anwar M., Abdullah A. (2014). Importance Of Cervical Screening In Women. J. Nurs..

[290] Kitchener H.C., Gilham C., Sargent A., Bailey A., Albrow R., Roberts C., Desai M., Mather J., Turner A., Moss S. (2011). A comparison of HPV DNA testing and liquid based cytology over three rounds of primary cervical screening: Extended follow up in the ARTISTIC trial. Eur. J. Cancer.

[291] Hilton S., Hunt K., Langan M., Bedford H., Petticrew M. (2010). Newsprint media representations of the introduction of the HPV vaccination programme for cervical cancer prevention in the UK (2005–2008). Soc. Sci. Med..

[292] Smith J.H.F., Herrington C.S. (2017). Cervical Screening: History, Current Algorithms, and Future Directions. Pathology of the Cervix.

[293] Williams D., Davies M., Fiander A., Farewell D., Hillier S., Brain K. (2017). Women’s perspectives on human papillomavirus self-sampling in the context of the UK cervical screening programme. Health Expect..

[294] Westre B., Giske A., Guttormsen H., Sørbye S.W., Skjeldestad F.E. (2016). 5-type HPV mRNA versus 14-type HPV DNA test: Test performance, over-diagnosis and overtreatment in triage of women with minor cervical lesions. BMC Clin. Pathol..

[295] Leinonen M.K., Campbell S., Ursin G., Tropé A., Nygård M. (2017). Barriers to cervical cancer screening faced by immigrants: A registry-based study of 1.4 million women in Norway. Eur. J. Public Health.

[296] Nygård J.F., Skare G.B., Thoresen S.Ø. (2002). The cervical cancer screening programme in Norway, 1992–2000: Changes in Pap smear coverage and incidence of cervical cancer. J. Med. Screen..

[297] Haldorsen T., Skare G.B., Ursin G., Bjørge T. (2015). Results of delayed triage by HPV testing and cytology in the Norwegian Cervical Cancer Screening Programme. Acta Oncol..

[298] Sørbye S.W., Suhrke P., Revå B.W., Berland J., Maurseth R.J., Al-Shibli K. (2017). Accuracy of cervical cytology: Comparison of diagnoses of 100 Pap smears read by four pathologists at three hospitals in Norway. BMC Clin. Pathol..

[299] Engesæter B., van Diermen Hidle B., Hansen M., Moltu P., Staby K.M., Borchgrevink-Persen S., Vintermyr O.K., Lönnberg S., Nygård M., Janssen E.A.M. (2016). Quality assurance of human papillomavirus (HPV) testing in the implementation of HPV primary screening in Norway: An inter-laboratory reproducibility study. BMC Infect. Dis..

[300] Caspi R., Schejter E., Groutz A. (2016). Screening for Cervical Cancer Among Low-Risk Populations: Orthodox Jewish Women as a Model. J. Women’s Health.

[301] Bassal R., Schejter E., Bachar R., Shapira H., Sandbank J., Supino Rosin L., Schvimer M., Cohen D., Keinan-Boker L. (2014). Cervical Pap screening among Israeli women, 2005–2010. Arch. Gynecol. Obstet..

[302] Bassal R., Schejter E., Bachar R., Perri T., Korach J., Jakobson-Setton A., Ben-David L.H., Cohen D., Keinan-Boker L. (2016). Risk Factors for Cervical Cancer and CIN3 in Jewish Women in Israel—Two Case Control Studies. Asian Pac. J. Cancer Prev. APJCP.

[303] Amir H., Gophen R., Amir Levy Y., Hasson J., Gordon D., Amit A., Azem F. (2015). Obstetricians and gynecologists: Which characteristics do Israeli lesbians prefer?. J. Obstet. Gynaecol. Res..

[304] Lurie S., Mizrachi Y., Chodick G., Katz R., Schejter E. (2017). Impact of quadrivalent human papillomavirus vaccine on genital warts in an opportunistic vaccination structure. Gynecol. Oncol..

[305] Vassilakos P., Petignat P., Boulvain M., Campana A. (2002). Primary screening for cervical cancer precursors by the combined use of liquid-based cytology, computer-assisted cytology and HPV DNA testing. Br. J. Cancer.

[306] Dobec M., Bannwart F., Kilgus S., Kaeppeli F., Cassinotti P. (2011). Human papillomavirus infection among women with cytological abnormalities in Switzerland investigated by an automated linear array genotyping test. J. Med. Virol..

[307] Viviano M., Catarino R., Jeannot E., Boulvain M., Malinverno M.U., Vassilakos P., Petignat P. (2017). Self-sampling to improve cervical cancer screening coverage in Switzerland: A randomised controlled trial. Br. J. Cancer.

[308] Burton-Jeangros C., Cullati S., Manor O., Courvoisier D.S., Bouchardy C., Guessous I. (2017). Cervical cancer screening in Switzerland: Cross-sectional trends (1992–2012) in social inequalities. Eur. J. Public Health.

[309] Fargnoli V., Petignat P., Burton-Jeangros C. (2015). To what extent will women accept HPV self-sampling for cervical cancer screening? A qualitative study conducted in Switzerland. Int. J. Women’s Health.

[310] Raineri I. (2015). Cervical Screening In Switzerland: The Opportunistic Approach. Cytopathology.

[311] Szucs T.D., Largeron N., Dedes K.J., Rafia R., Bénard S. (2008). Cost-effectiveness analysis of adding a quadrivalent HPV vaccine to the cervical cancer screening programme in Switzerland. Curr. Med. Res. Opin..

[312] Karasu A.F.G., Adanir I., Aydin S., Ilhan G.K., Ofli T. (2017). Nurses’ Knowledge and Opinions on HPV Vaccination: A Cross-Sectional Study from Istanbul. J. Cancer Educ..

[313] Demir L.S., Asuk N.A., Demir N.A. (2017). Screening for breast and cervix cancers in a rural part of Turkey. Biomed. Res..

[314] Ozlem A., Umit I. (2015). Comparative analysis of cervical cytology screening methods and staining protocols for detection rate and accurate interpretation of ASC-H: Data from a high-volume laboratory in Turkey. Diagn. Cytopathol..

[315] Gultekin M., Zayifoglu Karaca M., Kucukyildiz I., Dundar S., Boztas G., Semra Turan H., Hacikamiloglu E., Murtuza K., Keskinkilic B., Sencan I. (2018). Initial results of population based cervical cancer screening program using HPV testing in one million Turkish women. Int. J. Cancer.

[316] Kir G., Seneldir H., Cosan Sarbay B. (2018). The clinical performance of computer-assisted liquid-based cytology, primary hrHPV screening, and cotesting at a Turkish Tertiary Care Hospital. Diagn. Cytopathol..

[317] Erbarut Seven I., Mollamemisoglu H., Eren F. (2017). Reliability of reporting the presence of transformation zone material in Papanicolaou smears using an automated screening system. Cytopathology.

[318] Türkmen İ.Ç., Usubütün A., Çakir A., Aydin Ö., Bolat F.A., Akbulut M., Altinay S., Arici S., Aslan F., Astarci M. (2017). What does the Data of 354,725 Patients from Turkey Tell Us About Cervical Smear Epithelial Cell Abnormalities?-The Epithelial Cell Abnormality Rate is Increasing-Quality Control Studies and Corrective Activity are Musts. Turk. J. Pathol..

[319] Yanikkerem E., Koker G. (2014). Knowledge, attitudes, practices and barriers towards HPV vaccination among nurses in Turkey: A longitudinal study. Asian Pac. J. Cancer Prev. APJCP.

[320] Oz M., Cetinkaya N., Apaydin A., Korkmaz E., Bas S., Ozgu E., Gungor T. (2018). Awareness and Knowledge Levels of Turkish College Students about Human Papilloma Virus Infection and Vaccine Acceptance. J. Cancer Educ..

[321] Bal-Yılmaz H., Koniak-Griffin D. (2018). Knowledge, Behaviors, and Attitudes About Human Papilloma Virus Among Nursing Students in Izmir, Turkey. J. Cancer Educ..

[322] Dzhafer N. (2016). The programme “Stop and get checked”—An attempt to restore cancer screening in Bulgaria. Scripta Scientifica Salutis Publicae.

[323] Enerly E., Bonde J., Schee K., Pedersen H., Lönnberg S., Nygård M. (2016). Self-Sampling for Human Papillomavirus Testing among Non-Attenders Increases Attendance to the Norwegian Cervical Cancer Screening Programme. PLoS ONE.

[324] Tamalet C., Halfon P., Retraite L.L., Grob A., Leandri F.X., Heid P., Sancho-Garnier H., Piana L. (2016). Genotyping and follow-up of HR-HPV types detected by self-sampling in women from low socioeconomic groups not participating in regular cervical cancer screening in France. J. Clin. Virol..

[325] Garcia F., Barker B., Santos C., Brown E.M., Nuño T., Giuliano A., Davis J. (2003). Cross-sectional study of patient- and physician-collected cervical cytology and human papillomavirus. Obstet. Gynecol..

[326] Szarewski A., Cadman L., Mesher D., Austin J., Ashdown-Barr L., Edwards R., Lyons D., Walker J., Christison J., Frater A. (2011). HPV self-sampling as an alternative strategy in non-attenders for cervical screening—A randomised controlled trial. Br. J. Cancer.

[327] McSherry L.A., Dombrowski S.U., Francis J.J., Murphy J., Martin C.M., O’Leary J.J., Sharp L. (2012). ‘It’s a can of worms’: Understanding primary care practitioners’ behaviours in relation to HPV using the theoretical domains framework. Implement. Sci..

